# Development of Nd (III)-Based Terahertz Absorbers Revealing Temperature Dependent Near-Infrared Luminescence

**DOI:** 10.3390/ijms23116051

**Published:** 2022-05-27

**Authors:** Kunal Kumar, Olaf Stefanczyk, Koji Nakabayashi, Yuuki Mineo, Shin-ichi Ohkoshi

**Affiliations:** Department of Chemistry, School of Science, The University of Tokyo, 7-3-1 Hongo, Bunkyo-ku, Tokyo 113-0033, Japan; kunal-k@chem.s.u-tokyo.ac.jp (K.K.); olaf@chem.s.u-tokyo.ac.jp (O.S.); knakabayashi@chem.s.u-tokyo.ac.jp (K.N.); mineo-y@chem.s.u-tokyo.ac.jp (Y.M.)

**Keywords:** terahertz time-domain spectroscopy, sub-terahertz Raman, ratiometric thermometer, ab initio calculation, luminescence, optical properties

## Abstract

Molecular vibrations in the solid-state, detectable in the terahertz (THz) region, are the subject of research to further develop THz technologies. To observe such vibrations in terahertz time-domain spectroscopy (THz-TDS) and low-frequency (LF) Raman spectroscopy, two supramolecular assemblies with the formula [Nd^III^ (phen)_3_ (NCX)_3_] 0.3EtOH (X = S, **1**-**S**; Se, **1**-**Se**) were designed and prepared. Both compounds show several THz-TDS and LF-Raman peaks in the sub-THz range, with the lowest frequencies of 0.65 and 0.59 THz for **1**-**S** and **1**-**Se**, and 0.75 and 0.61 THz for **1**-**S** and **1**-**Se**, respectively. The peak redshift was observed due to the substitution of SCN^−^ by SeCN^−^. Additionally, temperature-dependent TDS-THz studies showed a thermal blueshift phenomenon, as the peak position shifted to 0.68 THz for **1-S** and 0.62 THz for **1**-**Se** at 10 K. Based on ab initio calculations, sub-THz vibrations were ascribed to the swaying of the three thiocyanate/selenocyanate. Moreover, both samples exhibited near-infrared (NIR) emission from Nd (III), and very good thermometric properties in the 300–150 K range, comparable to neodymium (III) oxide-based thermometers and higher than previously reported complexes. Moreover, the temperature dependence of fluorescence and THz spectroscopy analysis showed that the reduction in anharmonic thermal vibrations leads to a significant increase in the intensity and a reduction in the width of the emission and LF absorption peaks. These studies provide the basis for developing new routes to adjust the LF vibrational absorption.

## 1. Introduction

Molecular materials are attractive to researchers due to their simple structures and predisposition to integrate various properties, such as magnetism [1,2,3,4] (e.g., long-range magnetic ordering [5,6,7,8,9], spin crossover [10], etc.), sorption [11,12,13], luminescence [14,15], conductivity [16,17], non-linear optics [18,19,20], and low frequency absorbing capability [21,22,23]. The judicious selection of building blocks and their arrangements in solid-state structures results in the desired behaviors of molecular origin. Therefore, the required functionality can be implemented at a very early stage of design. Up to now, a variety of multifunctional soft materials based on metal-organic compounds have been prepared [24,25,26,27].

Low-frequency (LF) absorption in the terahertz (THz) region is usually associated with molecular vibrations in the crystal structure and can be detected by terahertz time-domain spectroscopy (THz-TDS) and LF-Raman spectroscopy. Surprisingly, LF absorption research has not received enough attention, despite its great potential for application [28,29,30,31,32]. LF absorbing materials can lead to a breakthrough in THz technologies used in medicine for illegal substance detection, gas sensing in the event of accidents at fire sites or chemical plants, and quality checking of coated magnetic films or paints [33,34]. Furthermore, sub-THz detecting materials will be revolutionary in the development of next-generation wireless communication devices (beyond the fifth-generation 5G technology standard), as well as in the reduction in electromagnetic radiation pollution [35,36,37].

Considering the above-mentioned trends in advanced material research, lanthanide ions (Ln (III)) appear to be very promising metal centers for the construction of multifunctional molecular complexes, particularly given their intrinsic optical properties. Shielded f-orbitals of Ln (III) complexes enable a sharp electronic transition in the range from the ultraviolet (UV) and visible light (Vis) region to the near-infrared (NIR) region [38,39]. Emission in the NIR region has been fruitfully utilized for the development of luminescent molecular thermometers by analyzing the thermal evolution of the peak intensity and position, band shape, and lifetime with temperature [40,41,42,43]. The development of such emissive thermometers paves the way for accurate and non-contact thermometry, which is important in the field of medicine as well as for nanoscale electronic and photonic devices [44,45,46].

Notably, a limited number of studies on the THz-TDS and LF-Raman spectroscopies of Ln (III)-based assemblies and other transition metal compounds have been conducted [47,48,49]. Until now, only one strategy for producing sub-THz absorbing materials has been identified by utilizing heavy alkali metal ions (i.e., Rb^+^ and Cs^+^) trapped inside cyanido-bridged coordination polymers [21,22,23]. However, this method has limitations, due to the necessity to design a material based on a high-dimensional coordination polymer containing heavy ions, which is difficult to achieve for this class of materials [50]. Therefore, there is a great need to develop alternative methods of synthesis using much simpler complexes, which can additionally be easily modified.

In the present study, supramolecular assemblies based on the NIR-emissive lanthanide (III) complexes with linear pseudohalogens were designated as promising candidates to reveal LF light absorption. Furthermore, the impact of the substitution of the lighter element (S) for the heavier one (Se) in the pseudohalide anion (XCN^−^ on the shift of the LF vibrational modes was considered. Additionally, a temperature-dependent study of LF phonons along with luminescence was performed to determine the effect of cooling on the spectroscopic properties. Consequently, two isostructural crystalline materials with the molecular formula [Nd^III^ (phen)_3_ (NCX)_3_] 0.3EtOH (X = S, **1**-**S**; Se, **1**-**Se**), containing three 1,10-phenanthrolines (phen) and three thiocyanates/selenocyanates with Nd (III) as central ions, were synthesized from an ethanol solution. Both compounds were comprehensively characterized with THz-TDS and Raman spectroscopies to unravel the nature of vibrations present in the low-frequency region. These vibrations were further assigned with ab initio calculations. Finally, both complexes were investigated for their luminescence-based thermometric properties.

## 2. Results and Discussion

### 2.1. Crystal Structure

**Figure 1:**
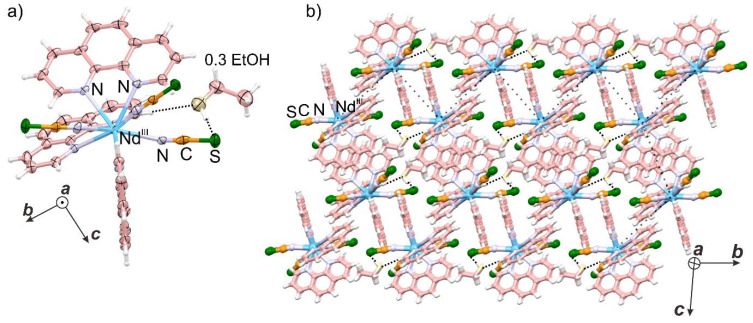
The crystal structure of **1**-**S**: (**a**) the anisotropic unit with atomic labeling; (**b**) the representative crystal packing along the *bc*-crystallographic plane. The complex **1**-**Se** has similar crystal packing. The densely dotted lines correspond to hydrogen bonding, and sparsely dotted zig-zag connections indicate the packing arrangement of Nd (III) ions.

The compounds **1**-**S** and **1**-**Se** crystallize in the centrosymmetric triclinic *P*-1 space group (Figure 1 and Figure S1, as well as Tables S1–S3). The asymmetric units of both materials consist of nine-coordinated Nd (III) complexes, with three nitrogen atoms of thiocyanate/selenocyanate anions and six nitrogen atoms of three phen ligands (Figure 1a). They also contain partially occupied solvent molecules at room temperature, trapped between two mononuclear units through hydrogen-bonding. The continuous shape measure (CSM) analysis confirmed that the first coordination sphere of Nd (III) ions has an unusual muffin-shape geometry (Figure S1a and Table S3), with the N3, N6, and N8 atoms forming the basal trigonal plane, the N2, N4, N5, N7, and N9 atoms forming the distorted equatorial pentagonal plane, and the N1 atom located at the muffin’s vertex. Interestingly, one of the thiocyanate ligands takes part in each plane, while the rest of the nitrogen atoms come from the phen ligands. As a result of the above type of arrangement, two of the thiocyanate ligands occupy the pseudo axial side, and one lies on the equatorial site, resulting in the overall meridional arrangement of thiocyanates. The analysis of the bond parameters in the first coordination sphere reveals that the average distance between Nd (III)-N (phen) ligands (2.689 and 2.677 Å for **1**-**S** and **1**-**Se**, respectively) is larger than Nd (III)-N (thiocyanate/selenocyanate) (2.508 and 2.511 Å for **1**-**S** and **1**-**Se**, respectively). Moreover, the distribution of bond distance for **1**-**S** and **1**-**Se** is relatively small (Table S2), illustrating strong bonding between thiocyanate/selenocyanate and Nd^3+^ ions. The shortest bond between Nd (III)–Nd (III) ions is 9.379 Å. The structure is stabilized by the hydrogen bonding between ethanol and sulfur atoms, as well as by the bonding of hydrogen from an aromatic ring of phen with the oxygen of ethanol (Figure 1). Additionally, the most common interaction is between the aromatic rings of phen via the π-π interaction, with approximately 3.7 Å centroid···centroid distance. Because these rings are stacked in the offset stacking fashion along with all three directions, these supramolecular arrangements are stable even after the partial removal of the crystallization solvent (Figure 1 and Figure S1). Along the *b*- and *c*-crystallographic axes, the two neighboring layers of the mononuclear unit form zig-zag arrangements (Figure 1b). Furthermore, the elemental compositions were confirmed by the thermogravimetric analyses and elemental analyses (Figure S2). Moreover, the purity and crystallinity of the powdered samples of **1**-**S** and **1**-**Se** were established by comparing the diffraction patterns simulated from single-crystal X-ray diffraction (SXRD) and powder X-ray diffraction (PXRD) data, which display sharp and non-split peaks (Figure S3). To corroborate the presence of pseudohalogens and phen vibrational modes, infrared (IR) spectra in the range of 3500–400 cm^−1^ and Raman spectra in the range of 3500–100 cm^−1^ were measured (Figures S4 and S5). Both vibrational spectroscopies affirmed the absence of ethanol-related peaks usual for such compounds [51].

### 2.2. Vibrational Signature

**Figure 2:**
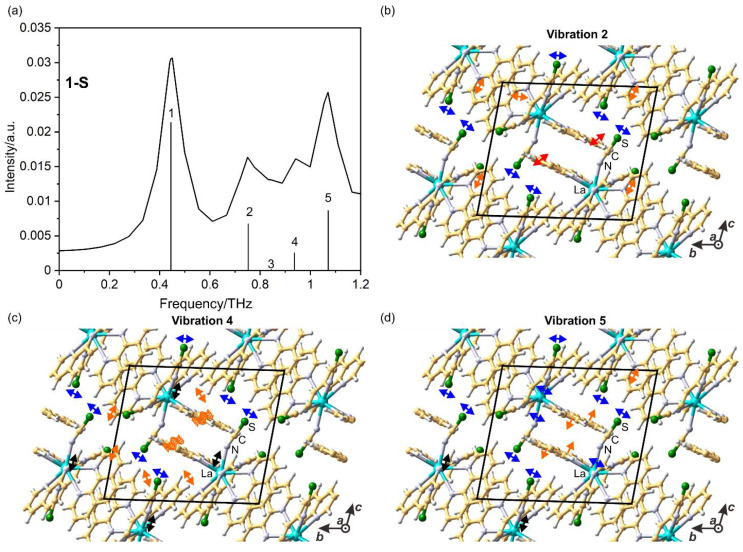
(**a**) Calculated vibrational IR active modes, and (**b**–**d**) graphic representations of the lowest energy phonon modes: 2, 4, and 5, for **1-S**. The blue, black, and orange arrows indicate the vibration vectors. The numbers on top of the bars in (**a**) correspond to the vibrations in (**b**–**d**), and the height of these bars represents their calculated relative intensity. Graphical representations of vibrations 1 and 3 have been omitted due to the imaginary nature of the first peak and the low intensity of the second one.

**Figure 3:**
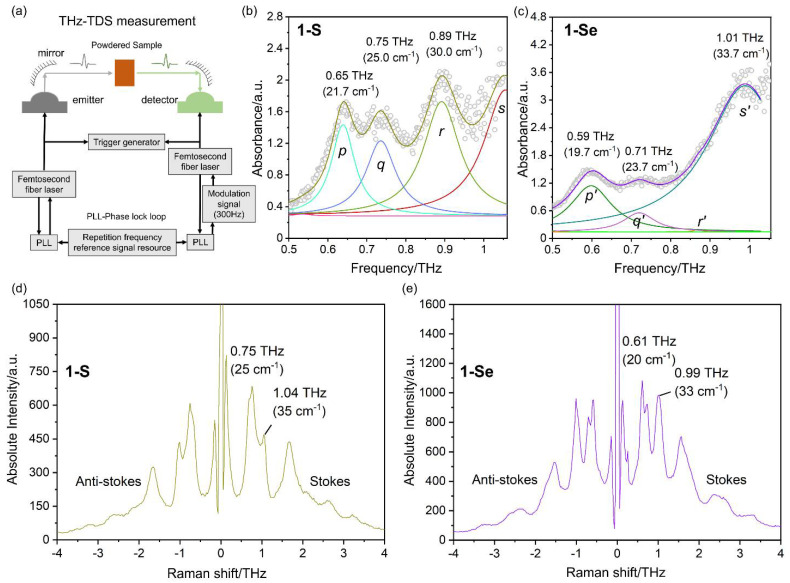
(**a**) Schematic diagram of the THz-TDS measurement setup used in experiments; (**b**,**c**) fill ratio normalized THz absorption spectrum of **1-S** and **1-Se** in the 0.5–1.05 THz region with deconvoluted peaks. (**d**,**e**) The LF-Raman shifts are presented for **1-S** and **1-Se** in the −4-4 THz region with anti-Stokes and Stokes type scattering peaks. The peaks in (**b**,**c**) were deconvoluted with the Lorentzian function into four different components: *p* (cyan), *q* (light blue), *r* (light green), and *s* (deep red) for **1-S** and *p*’ (deep green), *q*’ (magenta), *r*’ (orange), and *s*’ (turquoise) for **1-Se**. The circles in (**b**,**c**) represent the experimental THz absorption, along with solid yellow-green (**1-S**) and deep magenta (**1-Se**) lines depicting the simulated curves.

The simulated IR and Raman spectra for both complexes using density functional theory (DFT) showed good agreement with the experimental spectra in the high-frequency region, with a slight shift due to computational approximations (Figures S4 and S5). The relevant assignments corresponding to the several vibrational modes of pseudohalides and phen ligands are listed in Table S4. It also predicted several Raman and IR active vibrational modes in the sub-THz region with relatively high intensity compared to the (*ν* (C ≡ N)) vibrations associated with the combined motion of thiocyanate and phen ligands (Figure 2). Subsequently, THz-TDS absorption and LF-Raman scattering measurements on the powdered and single crystals were performed to detect both types of phonon modes (Figure 3 and Figures S6–S8). In this work, THz-TDS spectra are collected in the transmission mode on two different modules of the Advantest TAS7400TS, working in the 0.03–2 THz and 0.5–7.0 THz frequency range. The reproducibility of peaks for the THz spectra was validated with different experiments by increasing the thickness and weight of the powdered sample (Figure S6). It should be noted that **1**-**S** has three broad peaks centered around 0.65 THz (21.7 cm^−1^; *p*), 0.75 THz (25.0 cm^−1^; *q*), and 0.89 THz (30.0 cm^−1^; *r*), which shifts in the low frequency region for the first two vibrations upon heavier element substitution in complex **1**-**Se** and is positioned at 0.59 THz (19.7 cm^−1^; *p*’), 0.71 THz (23.7 cm^−1^; *q*’), and 1.01 THz (33.7 cm^−1^; *r*’) in the sub-THz region (Figure 3b,c). These broad vibrational peaks observed at room temperature are correlated with calculated vibrations 2–5 (Figure 2). An absorption plot normalized based on the fill ratio (Figure 3b,c) confirms that **1-Se** has higher absorption than **1-S.** Above 1 THz, several vibrational modes are also observed, which exhibit redshift upon selenium replacement. Interestingly, the observed phonons in the 1–3 THz region show different relative intensities for both assemblies. Sample **1**-**S** has the broad and indistinguishable feature of peaks in the 1–2 THz region; on the other hand, compound **1**-**Se** has a relatively sharp feature in this domain. However, it exhibits broad and indistinct features in the 2–3 THz spectral zone (Figure S6). Similarly, LF-Raman spectra monitored with Stokes and anti-Stokes scattering exhibited sharp peaks in the sub-THz region for single crystals of both assemblies. These peaks are located at 0.75 THz (25.0 cm^−1^) and 1.04 THz (35.0 cm^−1^) for **1**-**S,** and 0.61 THz (20.0 cm^−1^) and 0.99 THz (33.0 cm^−1^) for **1**-**Se** (Figure 3d,e). A similar redshift of the observed phonon energies is observed in the Raman scattering upon replacement with heavier selenium elements. The novelty of these Raman shifts in the complex can also be judged by looking at Raman scattering patterns of pure phen ligand, which yield minimum vibrations of 1.08 THz (36.0 cm^−1^) (Figure S7).

**Figure 4:**
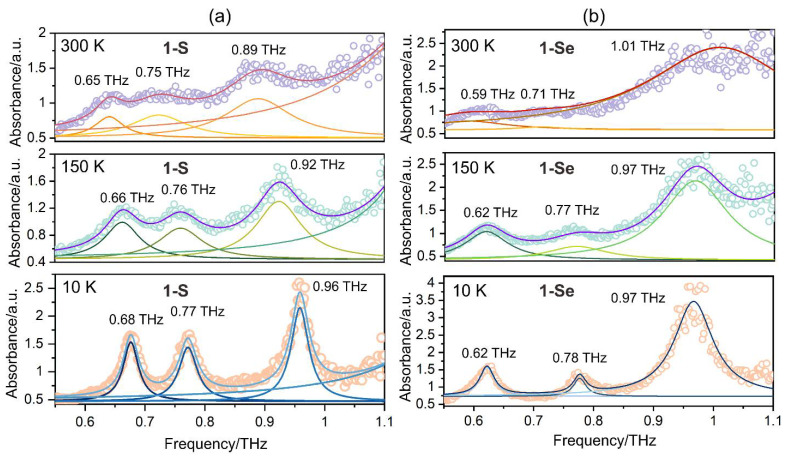
(**a**,**b**) THz-TDS spectra of **1-S** and **1-Se** measured at 300, 150, and 10 K. Each spectrum is deconvoluted with the Lorentzian function. The circles indicate experimental data, and the solid line shows fitting functions. Simulated cumulative plots and Lorentzian components for 3 different phonons at 300, 150, and 10 K are represented as red/orange, violet/green, and blue/dark blue lines.

Furthermore, temperature-dependent THz-TDS spectroscopy in the range of 300–10 K was performed to reduce the anharmonic thermal vibrations included in the low-frequency THz peaks (Figure 4 and Figure S8). The lowering of the temperature had a positive effect on the reduction in the width of the peaks and a slight shift in their positions towards the higher energy region, which isa blueshift. At 10 K, 3 sharp peaks were observed around 0.68 THz (22.7 cm^−1^), 0.77 THz (25.7 cm^−1^), and 0.96 THz (32.0 cm^−1^) for **1-S**, and 0.62 THz (20.7 cm^−1^), 0.78 THz (26.0 cm^−1^), and 0.97 THz (32.3 cm^−1^) for **1-Se**. A similar peak width reduction effect upon cooling was previously observed for the coordination polymer Rb^I^ [Co^II^ (3-CNpy)_2_] [W^V^(CN)_8_], where 3-CNpy = 3-cyanopyridine [22]. In addition, the lowest energy peak in the sub-THz region, associated with the phonon mode of the Rb^+^ ion, revealed a blueshift during cooling below 150 K. Most probably, the blueshift is linked with the volume reduction in the crystal structure; however, this statement could not be unequivocally concluded based on the previously obtained results for Rb^I^ [Co^II^ (3-CNpy)_2_] [W^V^ (CN)_8_], due to the occurrence of a phase transition in this material. Therefore, the results for **1-S** and **1-Se**, showing no phase transitions, are a significant confirmation of this thesis. It is also worth emphasizing that at low temperatures, the **1-Se** sub-THz peaks maintain their redshift compared to the **1-S** peaks.

### 2.3. Quantum Chemical Calculation

To understand the nature of the low-frequency phonons, DFT calculations using Vienna Ab initio Simulation Package (VASP) software were performed by applying the periodic boundary conditions in the *k*-mesh of 3 × 3 × 3. Because of the computational cost and accuracy in solving initial geometry, **1-S** was used by replacing the Nd (III) ions in the asymmetric unit with La (III) ions. The calculated IR spectra contain five imaginary vibrations (Table S5). The fifth phonon has positive eigenvalues along the selected *k*-space (Figure S9). Close to zero negative frequency suggests that the geometry is already optimized. However, as the force accuracy criteria was not very low, imaginary frequency was observed. Therefore, the phonons are presented graphically, starting from the fifth vibration, which is referred to as the first vibration in Figure 2. The analysis of sharp low-frequency peaks in the THz and Raman spectra suggests their complex character, including both pseudohalide and organic ligand vibrations with respect to the Nd (III) position. (Figure 2, and Movies SV2–SV5). The packing effect is also visible through these simulations among the phen ligands governed by long-range interactions. Calculated peaks 2 (0.75 THz), 4 (0.936 THz), and 5 (1.071 THz) are approximately 100 times stronger than the peak 3 (0.844 THz) phonon, which reproduces the experimental observation with slight overestimation. These longitudinal phonon modes are both IR and Raman active. To understand the vibrations at high frequencies in both compounds, gas-phase simulations with Gaussian 16 are used [52].

### 2.4. Concluding Remark on Vibrational Features

Compounds **1-S** and **1-Se** are the first examples of complexes that exhibit experimentally-proven IR and Raman active modes in the sub-THz region. Moreover, the measured THz-TDS signals are the lowest energy among the materials tested so far, while the lowest energy Raman signals are similar to the values for [Yb^III^ (TPPO)_3_ (NCX)_3_], with the best characteristics among the available LF-Raman active systems (Table 1) [47]. Interestingly, discrete complexes with pseudohalogens seem to be the universal platform for constructing LF absorbing materials. Furthermore, **1-S** and **1-Se** show that the phonon energy of pseudohalogens’ swinging vibrations can be altered toward the low-frequency region (the redshift) upon increasing the weight of the involved atoms in the analogous structure. Notably, this effect contradicts the previous observations of blueshift in THz-TDS signals noted for 3D A^I^_x_Cu^II^_7_ [Mo^IV^ (CN)_8_]_3.5 + x/4_·nH_2_O networks [21]. Concurrently, it agrees with the redshift for A^I^ [Co^II^ (3-CNpy)_2_] [W^V^ (CN)_8_] layered material [22]. Moreover, the influence of factors other than the molecular weight of constituent atoms cannot be ruled out, and this requires further research. Therefore, two major effects are assumed to play a key role in the observed spectroscopic properties. The first effect is related to the ligand’s increased weight (NCS^−^/NCSe^−^), and the second is the variation introduced regarding long-range interactions and associated collective dynamics in the crystal packing [53]. The compared vibrational features in the LF region for IR and Raman active modes for various types of assemblies might suggest that lower-dimensional complexes have smaller phonon energy (Table 1). However, a more conclusive argument can be put forward in this regard by measuring the LF vibrational nature of one-dimensional material. It provides a suitable strategy for modulating the wave absorber limit for both IR and Raman active vibrational modes and for reaching close to the 300 GHz (0.3 THz) absorption required for the sixth generation (6G) technology.

### 2.5. Optical Spectroscopy

**Figure 5:**
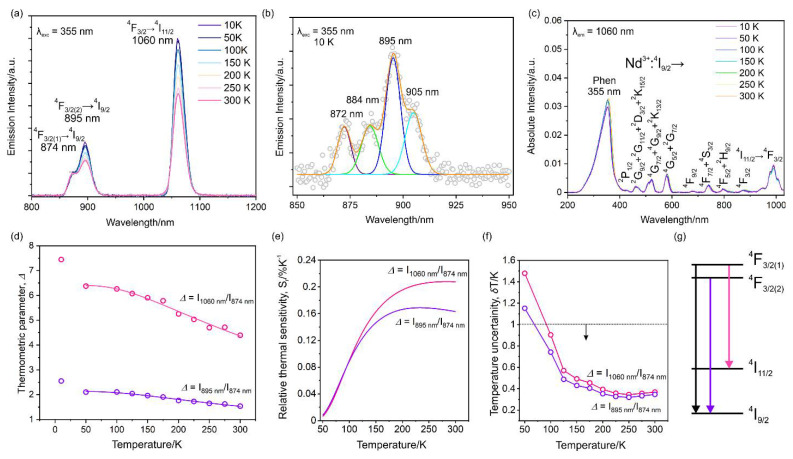
(**a**) Temperature-dependent emission spectra of **1-S**. (**b**) Deconvoluted high-resolution emission spectrum measured at 10 K for **1-S** with a 355 nm excitation light. (**c**) Temperature-dependent excitation spectra of **1-S** measured for a 1060 nm emissive peak. The circles for low-temperature spectra in Figure 5b are experimental data, and the solid orange line is a simulated line with deconvoluted four different constituents (brown, green, blue, and cyan) ascribable to the electronic transitions from the lowest state of the ^4^F_3/2_ multiplet to various states of the ^4^I_9/2_ multiplet. The full characterization of NIR-based emissive thermometry for **1-S** with the Δ being defined as the ratio between peak intensities, evaluated from emission intensities measured at a different temperature, (**d**) thermometric parameters in the 10−300 K range (magenta and olive circles) along with the fitted line in the range of 50−300 K, (**e**) the evaluated relative sensitivity at a different temperature from the fitted Δ curve, and (**f**) evaluated temperature uncertainty for different temperatures. (**g**) The energy level diagram of ground and excited multiplets involved in the emission.

The UV–Vis–NIR spectra measured at room temperature for the powder samples exhibit broad features in the high energy region around 330 nm for both compounds (Figure S10). However, the peak for **1-Se** is much broader than that for **1-S**. It is caused by the difference in the HOMO-LUMO gap, as well as by the involved frontier orbitals in the electronic transition. The HOMO orbitals are primarily concentrated on pseudohalide anions, and the LUMO orbitals involve phen ligands. Therefore, in the case of **1-Se**, selenocyanate destabilizes the HOMO orbitals more than thiocyanate, subsequently increasing the HOMO energy. In the NIR region predominantly, various direct transitions of Nd (III) ions are visible, which were assigned to transitions from the ground ^4^*I*_9/2_ state to various excited states [54]. The presence of direct excitation in absorption spectra prompted us to investigate its emission properties. Both **1-S** and **1-Se** reveal the NIR emission at about 895 nm (with shoulder centered at 874 nm) and 1060 nm (Figure 5 and Figure S11) upon excitation with UV, Vis, and NIR light. These emission peaks are ascribed to the radiative transitions from multiplets of ^4^*F*_3/2_ to ^4^*I*_9/2_ and ^4^*I*_11/2_ states, respectively. The measured excitation spectra for the emission wavelengths of 895 and 1060 nm show a similar appearance to the UV–Vis spectra, with 1 broad peak centered around 355 nm for **1-S** (364 nm for **1-Se**) linked with pseudohalides to phen transitions and several direct excitation peaks emanating from the Nd (III) centers in the visible and NIR region (Figure 5c, Figures S11c and S12). The NIR luminescence can also be observed by the direct excitation of Nd (III) at 520, 585, 740, and 800 nm for **1-S** and **1-Se** (Figure S13). The excitation by UV light results in emission due to energy transfer from the organic ligand to the central neodymium (III) ions.

The emission peaks at 895 nm and 1060 nm demonstrate an increase in their intensity with decreasing temperature for both supramolecular assemblies, which can be explained by the thermal cooling of vibrations located in the low-frequency region matching with ground-state energy, which in turn deactivates the non-radiative relaxation. This conclusion is supported by the results of temperature-dependent THz-TDS studies, suggesting a reduction in anharmonic thermal vibrations after cooling and is observed as a decrease in full width at half the maximum of individual peaks (Figure 4). The emission spectrum at 895 nm splits into four distinct peaks, with several other transitions overlapping into one peak at 10 K (Figure 5b and Figure S11b). The first two peaks are separated by 155.7 and 142.9 cm^−1^, respectively, for **1-S** and **1-Se**. To precisely determine the energy splitting of the Nd (III)-^4^*I*_9/2_ five ground multiplet, the emissive peaks should be deconvoluted into 10 different peaks, resulting in a separation of the first 2 states below 100 cm^−1^. Nonetheless, it shows that the distribution of these ground multiplets is different for **1-S** and **1-Se**. This was also confirmed by ab initio calculation using OpenMOLCAS (Tables S6 and S7) [55]. Detailed thermometric characterizations for the two complexes have been carried out by defining the thermometric parameter (Δ *(T)*) as the ratio between peaks positioned near 895 nm to 874 nm ([(I (^4^*F*_3/2(2)_ → ^4^*I*_9/2_)/I (^4^*F*_3/2(1)_ → ^4^*I*_9/2_)]) and 1060 nm to 874 nm ([(I (^4^*F*_3/2(1)_ → ^4^*I*_11/2_)/I (^4^*F*_3/2(1)_ → ^4^*I*_9/2_)]). Single-center ratiometric thermometer parameters are fitted with the Boltzmann depopulation equation using Equation (1).

Δ(*T*) = *A* + Δ_0_exp(−Δ*E_0_*/*k*_B_*T*) (1)

where *A* is the offset factor in kelvin, Δ_0_ is the pre-exponential factor in kelvin, Δ*E*_0_ is the energy gap of emissive levels at a given temperature, *k*_B_ is the Boltzmann constant, and *T* is the absolute temperature in kelvin.

The fitted parameters are listed in Table S8. The performance of both thermometric parameters decreases upon increasing temperature for **1-S** and **1-Se**. To further characterize the working suitability, sensitivity (*S*_r_) and uncertainty (*δT*) are computed using Equations (2) and (3).

(2) $$S_{r}=\frac{1}{\Delta }\left(\frac{\partial \Delta }{\partial T}\right)\times 100$$

(3) $$\delta T=\left(\frac{\delta \Delta }{\Delta }\right)/S_{r}$$

The observed maximum sensitivity is 0.207% K^−1^ for **1-S,** corresponding to Δ (1060/874) at 300 K and 0.207% K^−1^ for **1-Se** at 170 K related to Δ (895/874). These sensitivity parameters are comparable to oxide-based thermometers in which the Nd^3+^ ion is embedded in various oxides and they are higher than previously reported complexes [56,57]. The transition Δ (1060/874) emanates from the non-coupled ^4^*F*_3/2(1)_ excited level, unlike other thermometric parameters, as a result of which the Mott–Seitz model (Equation (S1)) is also used to examine the thermometric data (Figure 5g, Tables S6 and S7). It retains a similar sensitivity range, with a maximum of around 0.207% K^−1^ for **1-S** and 0.295% K^−1^ for **1-Se** at 200 and 50 K, respectively (Figure S14). Temperature uncertainty is below 1 K at high temperatures (above 100 K) for the two compounds (Table S9). The repeatability of the thermometric parameters was also confirmed in three cycles, exhibiting reliability of thermometric performance (Figure S15). Overall, **1-S** and **1-Se** show the best performance at sensing temperature in the high-temperature range of 150–300 K.

## 3. Materials and Methods

### 3.1. Materials

Neodymium (III) chloride hexahydrate (CAS: 13477-89-9, Sigma-Aldrich, St. Louis, MI, USA), 1,10-phenanthroline monohydrate, phen (CAS: 791-28-6, Wako Pure Chemicals Industries, Ltd., Osaka, Japan), potassium thiocyanate, KSCN (CAS: 333-20-0, Wako Pure Chemicals Industries, Ltd.) and potassium selenocyanate, KSeCN (CAS: 3425-46-5) were obtained and used without further purification.

### 3.2. Synthetic Procedure of [Nd^III^ (phen)_3_ (NCX)_3_]·0.3EtOH Complexes (1-S and 1-Se)

A total of 5 mL of 0.05 M ethanol solution of NdCl_3_·6H_2_O was mixed with 5 mL of 0.16 M ethanol solution of KSCN/KSeCN. The resulting mixture was stirred for 30 min, resulting in a white precipitate of KCl. On top of the filtered solution, a 0.16 M (5 mL) solution of phen was added slowly, resulting in rapid crystallization. The needle-shaped crystals were obtained and dried. Light blue crystals of **1-S** and light pink crystals of **1-Se** were obtained. To avoid the formation of brown colloidal selenium, **1-Se** synthesis was carried out in a glovebox; however, post-crystallization actions, such as filtration and drying, were carried out in the air. Single-crystal X-ray diffraction (SXRD) measurements confirmed the molecular structure of both complexes. The yield of **1-S** was 187 mg (85% based on Nd (III) content). Anal. calcd for NdS_3_C_39.66_H_26_N_9_O_0.33_ (*M*_W_ = 875.1 g/mol, **1-S**): Nd, 16.49%; S, 11.00%; C, 54.48%; H, 3.00%; N, 14.42%. Found: Nd, 16.50%; S, 10.93%; C, 54.14%; H, 3.07%; N, 14.58%. FT-IR of **1-S** (in KBr matrix, cm^−1^): 2140s, 2158s (broad) (*ν* (C ≡ N)). FT-Raman of **1-S** (single crystal, cm^−1^): 2140s, 2163s (sharp) (*ν* (C ≡ N)). Yield **1-Se**: 224 mg (88% based on Er). Anal. calcd for NdSe_3_C_39.66_H_26_N_9_O_0.33_ (*M*_W_ = 1015.8 g/mol, **1-Se**): Nd, 14.21%; C, 46.93%; H, 2.58%; N, 12.42%. Found: Nd, 14.41%; C, 46.55%; H, 2.69%; N, 12.29%. FT-IR of **1-Se** (in KBr matrix, cm^−1^): 2141s, 2155s (broad) [*ν* (C ≡ N)]. FT-Raman of **1-Se** (single crystal, cm^−1^): 2141s, 2157s (sharp) (*ν* (C ≡ N)).

### 3.3. X-ray Crystallography

Single-crystal X-ray diffraction (SXRD) measurements of **1-S** and **1-Se** were performed using a Rigaku AFC10 diffractometer equipped with a Rigaku Saturn Kappa CCD detector and a MicroMax-007 HF/VariMax rotating-anode X-ray generator with monochromated MoKα radiation. The SXRD measurement was performed on the crystals directly taken from the mother solution at 300 K, covered in a paratone N-oil, and mounted on a Micro Mounts holder. The diffraction data were reduced and integrated with the CrisAlisPro software of Rigaku. The crystal structure was solved by a direct method using SHELXS-97 and refined by a *F*^2^ full-matrix least-squares technique with the SHELXL-2014/7 program [58,59]. All further calculations were executed using the Olex 2-1.2 software integrated system [60]. For all non-hydrogen atoms, anisotropic refinement was performed while hydrogen atoms were placed using an electron density map. The summary of the crystal data and structure refinement are gathered in Supplementary Materials, Table S1. The additional crystallographic information for **1-S** and **1-Se** can be found in CCDC 2105914 and 2105915 records. The structural data in the figures were created using the CCDC Mercury 3.10 visualization software [61]. The geometries of metal centers were estimated with the continuous shape measures (CSM) analysis with the use of SHAPE v2.1 software [62,63].

### 3.4. Physical Techniques

Infrared absorption spectra of all the compounds dispersed in the KBr matrix were obtained with the help of a JASCO FTIR-4100 spectrometer. A JASCO NRS-7500 laser Raman spectrometer was utilized in reflection mode to perform Raman spectroscopy on the single crystals of **1-S** and **1-Se**. Elemental analyses of metal concentrations (Nd, S, and Se) were performed using an Agilent 7700 inductively coupled plasma mass spectrometer. C, H, and N analyses were performed on an Elementar Analysensysteme GmbH: vario MICRO cube. Powder X-ray diffraction (PXRD) patterns were recorded with a RIGAKU Ultima IV diffractometer, equipped with Cu Kα radiation (*λ* = 1.541 Å). Thermogravimetric analyses were measured using a Rigaku Thermo Plus TG8120 apparatus in the 20–400 °C range under an air atmosphere, with a 1 °C/min heating rate and Al_2_O_3_ as reference material. UV–Vis–NIR diffuse reflectance (absorption) spectra were collected using a JASCO V-670 spectrophotometer on the polycrystalline samples crushed on cellulose paper. The fluorescence properties were measured in the reflectance mode with the Horiba Jobin-Yvon Fluorolog^®^-3 (FL3-211) spectrofluorometer (model TKN-7), equipped with a Xe lamp (450 W) as an excitation source, and the R928P detector working in the photon counting mode. A powdered sample blocked with paraffin oil was sandwiched between two high-purity quartz plates placed inside a liquid helium-cooled microscopy cryostat (Oxford Instruments), which was connected to the digital temperature controller for thermal-dependent emission spectra measurement. The sweeping rate for thermal dependence luminescence experiments was maintained at 2 °C min^−1^. The THz absorption spectra of the polycrystalline samples were measured by a THz-TDS system of Advantest TAS7400TS, equipped with a Cherenkov type THz generator and emitter using a LiNbO_3_ waveguide and a 1550 nm fiber laser (50 fs, 150 mW; Figure 3). The THz pulse was condensed with silicon lenses and paraboloidal mirrors and was irradiated into the sample placed in a polyethylene cell. The electric fields of the transmitted THz pulse wave formed in the time domain were obtained, and the power spectrum was obtained by Fourier transformation. The measurement at 293 K was performed by forming a polycrystalline sample into a pellet with a variable thickness to confirm the validity of the peaks. For the temperature-dependent measurements, powder samples were sandwiched between two white polycarbonate plates and one aluminum plate with circular holes (Ø = 8 mm). The samples were placed in the circular hole closed in the middle with the help of a polycarbonate plate. The attached liquid helium cryostat was cooled with a cooling rate of 1 K/min.

### 3.5. Ab Initio Calculations

Structure optimizations were conducted using Gaussian 016 software for inputs consisting of the asymmetric crystallographic structures of **1-S** and **1-Se** measured at 300 K under the following two conditions: with and without solvent molecules (Figure 1) [52]. For optimization, the Perdew–Burke–Ernzerhof (PBE) [62] and frequency calculation Becke, 3-parameter, Lee–Yang–Parr (B3LYP) [64] density-functionals were utilized, along with Grimme’s D3 dispersion correction [65], the correlation-consistent polarized valence-only double-zeta (cc-pVDZ) basis set was employed for atoms not directly coordinated to lanthanide ions, and the correlation-consistent polarized valence-only triple-zeta (cc-pVTZ) basis set was used for nitrogen atoms in the first coordination sphere [66,67]. For closed-shell ab initio calculations, the Nd (III) ion was replaced by La (III), and the Stuttgart RSC 1997 effective core potential (ECP) was used with the 28 core electrons of lanthanum, and the remaining valence electrons were described with the corresponding valence basis set [68,69]. The frequency calculation after correction showed no negative vibration, suggesting that both compounds have reached a stationary point. Time-dependent density functional theory (TDFT) closed-shell calculations were carried out by setting the charge and multiplicity to ‘0 and ‘1’ with B3LYP functional and using the same basis set described above, using the first 100 lowest-lying excited states. Theoretical calculations using OpenMolcas 8 [55] were performed by combining the complete active space self-consistent field (CASSCF), restricted active space state-interaction (RASSI), and SINGLE_ANISO for the optimized geometry. Basis sets from the MOLCAS ANO-RCC library [70,71] were utilized, with triple-zeta valence plus polarization (VTZP) quality for the Nd (III) cation, double-zeta valence plus polarization (VDZP) quality for the nitrogen donors neighboring neodymium ions, and VDZ quality for all the remaining atoms. The spin-orbit coupling was calculated with the atomic mean-field integrals, and the two-electron integrals were Cholesky decomposed. In CASSCF, the active space was filled by the seven 4f orbitals, and three occupied electrons were implemented as the active space and mixed by spin-orbit coupling [72]. By using the SINGLE_ANISO CRYS command in the input file, the crystal field splitting parameters were obtained. To include the periodic boundary conditions in the solid-state density functional theory calculations, the Vienna Ab initio Simulation Package (VASP) software was utilized with the projector-augmented wave (PAW) potentials [73]. The periodic models were used with the PBE density functional [74], along with Grimme’s DFT-D3 dispersion corrections [65], including the Becke–Johnson damping function [75]. Using the initial solved structure from the SXRD measurement, **1-S** was fully optimized (lattice parameters and atomic positions). Next, the Nd (III) ion was replaced with a La (III) ion, and the partially occupied ethanol molecules were removed. The phonon package included with VASP was used to obtain vibrational spectra for the active infrared modes [76]. The wave functions were based on the plane wave with an energy cutoff of 520 eV and converged to a force accuracy of 0.01 eV/atom. A *k*-mesh of 3 × 3 × 3 was used for Brillouin zone sampling. The electronic iteration convergence was set to 1 × 10^−9^ eV. Gaussian smearing of 0.2 eV was used to smear the electron occupation.

## 4. Conclusions

Two new molecular assemblies [Nd^III^ (phen)_3_ (NCX)_3_]·0.3EtOH (X = S, **1-S**; Se, **1-Se**) were synthesized and characterized chemically and structurally. The simple molecular structure of these compounds enabled us to perform molecular modeling to predict their vibrational spectra in the wide energy domain. The calculated spectra showed relatively intense sub-THz absorption bands in the IR and Raman spectra, which were confirmed experimentally using both research techniques at room and cryogenic temperatures. The measured THz-TDS spectra in the sub-THz region consist of three broad peaks that redshift upon the replacement of lighter sulfur with heavier selenium. An analogous effect is also observed in the Raman scattering spectra. The combined results provided useful information about the nature of vibrations involved in the low-frequency region. The discovery of the redshift of peaks by increasing the mass of elements is a significant contribution to the next decade’s 6G information technology. This study provides a platform to tune the LF THz absorption, as well as LF-Raman scattering, by increasing the bulkiness of the coordination environment around central metal ions. In the future, such complexes will be able to shift the THz absorption below 0.6 THz. Additionally, they show Nd (III)-centered NIR emission properties upon UV, visible, and NIR excitation. UV light primarily excites the organic ligand, which transfers its energy to the central lanthanide ion, while visible and NIR light directly excites the Nd (III) ion, resulting in NIR luminescence over a wide range of wavelengths. The NIR emission varies with temperature, ranging from 300–10 K, and can be utilized as a ratiometric temperature sensor.

## Figures and Tables

**Table 1:** List of compounds with the lowest frequency dominant THz-TDS and Raman signals.

**THz-TDS Absorption**			
**Compound**	**Structure**	**The Lowest Frequency Dominant Signal**	**Reference**
[Nd^III^ (phen)_3_ (NCS)_3_] 0.3EtOH (1-S)	Discrete complex	0.65 THz (*ν* ≈ 21.7 cm^−1^) @300 K	This work
		0.68 THz (*ν* = 22.7 cm^−1^) @10 K	
[Nd^III^ (phen)_3_ (NCSe)_3_]·0.3EtOH (1-Se)	Discrete complex	0.59 THz (*ν* ≈ 19.7 cm^−1^) @300 K	This work
		0.62 THz (*ν* ≈ 20.7 cm^−1^) @10 K	
Rb^I^ [Co^II^ (3–CNpy)_2_] [W^V^ (CN)_8_]	Layers with Rb^+^ between	0.78 THz (*ν* ≈ 26.0 cm^−1^) @300 K	[22]
		0.87 THz (*ν* ≈ 29.0 cm^−1^) @10 K	
Cs^I^ [Co^II^ (3–CNpy)_2_] [W^V^ (CN)_8_]	Layers with Cs^+^ between	0.69 THz (*ν* ≈ 23.0 cm^−1^) @300 K	[22]
Rb^I^_3/2_ Cu^II^_7_ [Mo^IV^ (CN)_8_]_31/8_·12H_2_O	Network with Rb^+^ inside	1.11 THz (*ν* ≈ 37.0 cm^−1^) @300 K	[21]
Cs^I^_2_Cu^II^_7_ [Mo^IV^ (CN)_8_]_4_·6H_2_O	Network with Cs^+^ inside	1.35 THz (*ν* ≈ 45.0 cm^−1^) @300 K	[21]
Cs_0.90_Mn [Fe (CN)_6_]_0.93_·1.9H_2_O	Network with Cs^+^ inside	1.40 THz (*ν* ≈ 46.7 cm^−1^) @300 K	[23]
**Raman Scattering**			
**Compound**	**Structure**	**The Lowest Frequency Dominant Signal**	**Reference**
[Nd^II I^(phen)_3_ (NCS)_3_]·0.3EtOH (1-S)	Discrete complex	0.75 THz (*ν* ≈ 25.0 cm^−1^) @300 K	This work
[Nd^III^ (phen)_3_ (NCSe)_3_]·0.3EtOH (1-Se)	Discrete complex	0.61 THz (*ν* ≈ 20.3 cm^−1^) @300 K	This work
[Yb^III^ (TPPO)_3_ (NCS)_3_]	Discrete complex	0.48 THz (*ν* ≈ 16.0 cm^−1^) @300 K	[47]
[Yb^III^ (TPPO)_3_ (NCSe)_3_]	Discrete complex	0.48 THz (*ν* ≈ 16.0 cm^−1^) @300 K	[47]

3-CNpy = 3-cyanopyridine; phen = 1,10-phenanthroline; TPPO = triphenylphosphine oxide.

## Data Availability

The data presented in this study are available on request from the corresponding author.

## Supplementary Materials

The following supporting information can be downloaded at: https://www.mdpi.com/article/10.3390/ijms23116051/s1.
