# Supplementary material for: Development of Nd (III)-Based Terahertz Absorbers Revealing Temperature Dependent Near-Infrared Luminescence
Source: Int J Mol Sci. 2022 May 27;23(11):6051. doi: 10.3390/ijms23116051 (PMC9181137; doi:10.3390/ijms23116051)
Supplement: Supplementary file 1 [file ijms-23-06051-s001.zip › Supplementary Materials.pdf]

Supplementary Materials

Development of Nd (III)-based Terahertz Absorbers Revealing Temperature Dependent Near-infrared Luminescence

Kunal Kumar,† Olaf Stefanczyk,† Koji Nakabayashi,† Yuuki Mineo† and Shin-ichi Ohkoshi*,†

† Department of Chemistry, School of Science, The University of Tokyo,
7–3–1 Hongo, Bunkyo–ku, Tokyo 113–0033, Japan.
*Corresponding author: ohkoshi@chem.s.u-tokyo.ac.jp

LIST OF CONTENTS

Single-crystal X-ray diffraction (SXRD) studies 3

Figure S1. The muffin-shaped coordination geometry of the [NdN_9] moiety, the representative crystal packing of **1-S** along *ab*- and *ac*-crystallographic planes with dotted black lines indicating the short contacts with solvent and monomeric Nd(III) unit. The selenium-containing complex **1-Se** has similar crystal packing. 3

Table S1. Crystal data and structure refinement parameters for **1-S** and **1-Se**. 3

Table S2. Selected distances and angles of **1-S** and **1-Se**. 5

Table S3. Results of Continuous Shape Measure (CSM) analyses for the first coordination sphere of Nd(III) ion of **1-S** and **1-Se**. 6

Thermogravimetric analysis 6

Figure S2. Thermogravimetric plots for **1-S** and **1-Se** with indicated weight losses. 6

Powder X-ray diffraction (PXRD) studies 7

Figure S3. Experimental and calculated powder X-ray diffraction patterns of **1-S** and **1-Se**. The calculated PXRD patterns were simulated based on the structures determined in the SXRD experiments at 300 K. 7

Vibrational spectroscopy 7

Figure S4. Infrared (IR) absorption spectra in KBr matrix with assignment for **1-S** and **1-Se** compared with calculated IR spectra. DFT calculations were performed using Gaussian 16 for two models with and without ethanol. **Figure S4a** is supplemented by IR spectra determined from first-principal phonon calculation using VASP. 7

Figure S5. Experimental Raman scattering spectra for single crystals with assignment for **1-S** and **1-Se** compared with calculated Raman spectra. DFT calculations were performed using Gaussian 16 for two models with and without ethanol in the input. 8

Figure S6. THz-TDS spectra of **1-S** and **1-Se** measured with two different measurement modules for different fill rates. 8

Table S4. List of IR and Raman active vibrational modes for **1-S** and **1-Se** without ethanol solvent, including relevant assignment. 9

Figure S7. Experimental Raman scattering spectra for single crystals of phenanthroline in the low-frequency region of -6–6 THz (-200–200 cm^{-1}) with Stokes and anti-Stokes Raman scattering signals and the high-frequency region of 100–3500 cm^{-1}. 18

Figure S8. Temperature-dependent THz-TDS spectra of **1-S** and **1-Se** determined in the range of 300–10 K. 18
Figure S9. Total DOS of the **1-S** and partial phonon DOS of La, S, C, N, and H. 19
Table S5. Calculated optical phonon modes obtained through first-principles calculations of **1-S**. 20
UV-VIS spectroscopy 25
Figure S10. Room-temperature solid-state UV-Vis-NIR absorption (Kubelka-Munk function) spectra of **1-S** and **1-Se** in 200–1200 nm and400–1200 nm ranges with indicated assignments, the calculated UV-Vis spectra for **1-S** and **1-Se** with Nd(III) substituted by La(III) in 200–1200 nm range, and obtained corresponding frontier molecular orbitals. 25
Emission spectroscopy and thermometric properties 26
Figure S11. Temperature-dependent emission spectra of **1-Se**. Deconvoluted high-resolution emission spectrum measured at 10 K for **1-Se** with a 364 nm excitation light. Temperature-dependent excitation spectra of **1-Se** measured for emissive peaks located at 1060 nm. The full characterization of NIR based emissive thermometry for **1-Se** with the Δ being defined as the ratio between peak intensities, evaluated from emission intensities measured at a different temperature: thermometric parameters in the 10−300 K range along with the fitted line in the range of 50−300 K, the evaluated relative sensitivity at a different temperature from the fitted Δ curve, evaluated temperature uncertainty for different temperatures. .. 26
Figure S12. Temperature-dependent excitation spectra of **1-S** and**1-Se** recorded for emissive peak centered at 896 nm. 26
Figure S13. Excitation wavelength-dependent near-infrared emission spectra collected at 300 K for **1-S** and **1-Se**. 27
Figure S14. Thermometric parameters measured in the 10−300 K range along with the fitted line in the range of 50−300 K for peaks positioned near 1060 nm and 874 nm. Evaluated relative sensitivity at a different temperature from the fitted Δ curve. Evaluated temperature uncertainty for different temperatures. 27
Figure S15. Three cycles of repeatability data of thermometric parameters measured for three temperatures 50, 100, and 200 K for indicated thermometric parameters. 28
Table S6: CASSCF−SO results for the $^4F_{9/2}$ ground term of **1-S**. 28
Table S7: CASSCF−SO results for the $^4F_{9/2}$ ground term of **1-Se**. 28
Mott−Seitz model for two electronic transitions 28
Table S8. List of the fitting parameters from Δ vs. temperature plot for **1-S** and **1-Se** fitted with Equation 1 given in the manuscript and Equation S1 29
Table S9. The summary of maximum and minimum calculated relative thermal sensitivity, S_r, and temperature uncertainty, ΔT, for **1-S** and **1-Se** at the indicated temperature. 29
References to Supporting Information 29

Single-crystal X-ray diffraction (SXRD) studies

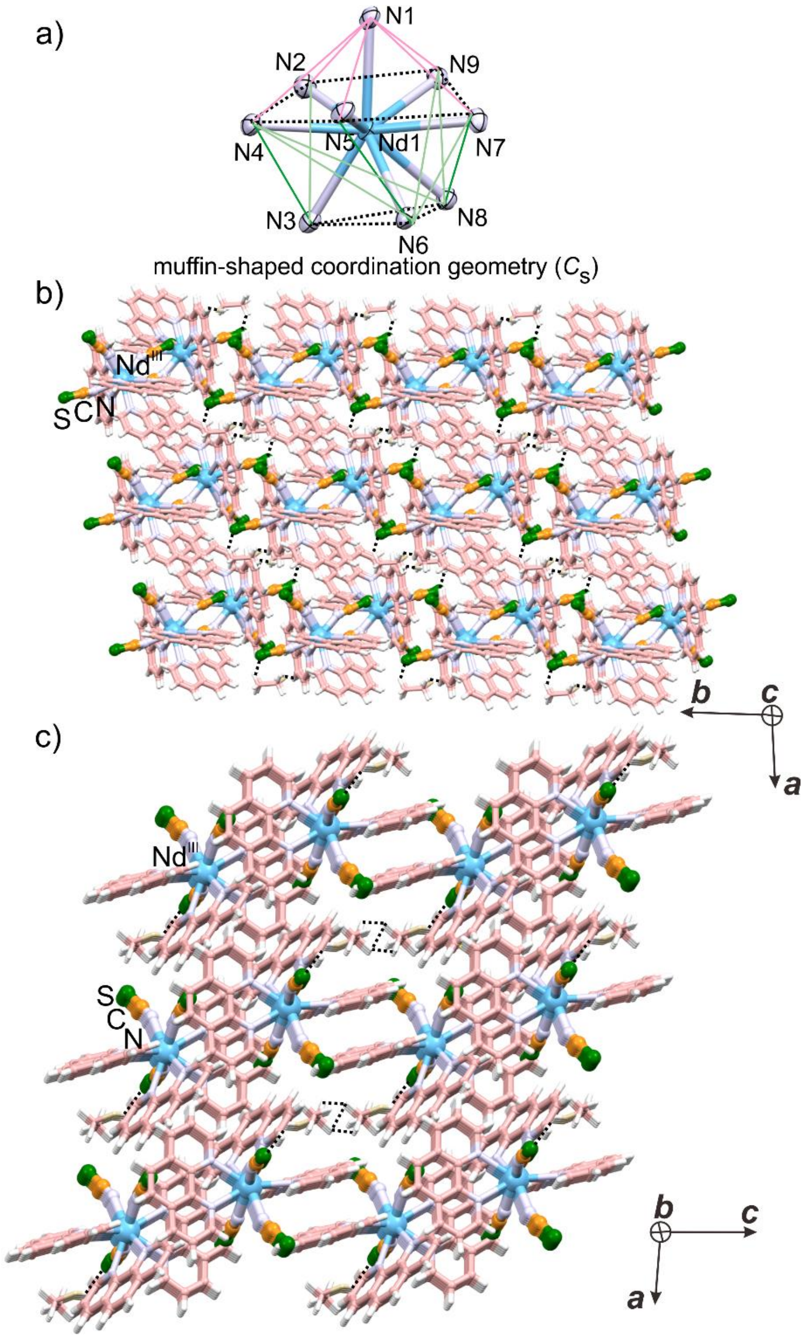

Figure S1. (a) The muffin-shaped coordination geometry of the [NdN_9] moiety, (b, c) the representative crystal packing of **1**-**S** along *ab*- and *ac*-crystallographic planes with dotted black lines indicating the short contacts with solvent and monomeric Nd(III) unit. The selenium-containing complex **1**-**Se** has similar crystal packing.

Table S1. Crystal data and structure refinement parameters for **1-S** and **1-Se**.

Compound		1-S	1-Se
**Formula**		$Nd1C_{39.66}H_{26}N_9O_{0.34}S_3$	$C_{39.66}H_{26}N_9NdO_{0.34}Se_3$
**Formula weight [g mol^{-1}]**		874.45	1015.15
***T* [K]**		293(2)	293(2)
**λ [Å]**		0.71073 (Mo Kα)	0.71073 (Mo Kα)
**Crystal system**		Triclinic	Triclinic
**Space group**		*P*-1	*P*-1
**Unit cell**	***a* [Å]**	10.9665(3)	11.1589(15)
	***b* [Å]**	12.2562(2)	12.4218(8)
	***c* [Å]**	15.3925(5)	15.5655(14)
	**α [°]**	97.193(2)	97.604(6)
	**β [°]**	101.718(3)	101.155(9)
	**γ [°]**	101.908(2)	102.185(8)
***V* [Å^3]**		1951.77(9)	2035.1(4)
Z		2	2
**Calcd. density**		1.488	1.657
**Abs. coefficient**		1.533	3.999
***F*(000)**		875.0	983.0
**Crystal size [mm^3]**		0.32 × 0.1 × 0.04	0.12 × 0.04 × 0.02
**Crystal morphology**		needle	needle
**2Θ range [°]**		3.45 to 54.792	3.836 to 54.898
**Limiting indices**		$-14 < h < 14$<br>$-15 < k < 15$<br>$-19 < l < 19$	$-14 < h < 14$<br>$-16 < k < 15$<br>$-19 < l < 19$
**Collected refls**		39779	37870
**Unique refls**		8210	8503
**R_{int}**		0.0615	0.1893
**Completeness**		100%	100%
**Data/restraints/parameters**		8210/12/498	8503/24/492
**GOF on F^2**		1.034	1.043
**Final *R* values**		R_1 = 0.0496 [I>2σ(I)]<br>wR_2 = 0.1325 [all]	R_1 = 0.1338 [I>2σ(I)]<br>wR_2 = 0.3365 [all]
**Largest diffr. peak/hole**		2.27/-0.64 e·A^{-3}	2.79/-1.41 e·A^{-3}

Table S2. Selected distances and angles of **1-S** and **1-Se**.

Parameter	Distance [Å]		Parameter	Distance [Å]	
	1-S	1-Se	1-S	1-S	1-Se
**Nd1–N1$_{(XCN)}$**	2.500(4)	2.500(16)	**Nd1–N4$_{(phen)}$**	2.686(4)	2.674(15)
**Nd1–N2$_{(XCN)}$**	2.489(4)	2.49(2)	**Nd1–N5$_{(phen)}$**	2.714(4)	2.692(16)
**Nd1–N3$_{(XCN)}$**	2.535(4)	2.543(14)	**Nd1–N6$_{(phen)}$**	2.660(3)	2.643(13)
			**Nd1–N7$_{(phen)}$**	2.665(4)	2.653(15)
			**Nd1–N8$_{(phen)}$**	2.706(3)	2.703(13)
			**Nd1–N9$_{(phen)}$**	2.708(4)	2.699(14)
**Parameter**	**Angle [°]**		**Parameter**	**Angle [°]**	
	**1-S**	**1-Se**	**1-S**	**1-S**	**1-Se**
**N1–Nd1–N3**	151.08(14)	151.0(5)	**N6–Nd1–N4**	100.37(11)	135.7(5)
**N1–Nd1–N4**	69.97(12)	69.7(5)	**N6–Nd1–N5**	65.99(11)	137.1(5)
**N1–Nd1–N5**	107.44(13)	108.2(5)	**N6–Nd1–N7**	61.35(11)	99.6(5)
**N1–Nd1–N6**	75.83(12)	75.6(5)	**N6–Nd1–N8**	69.83(11)	67.7(4)
**N1–Nd1–N7**	129.77(12)	130.3(5)	**N6–Nd1–N9**	120.62(11)	62.3(5)
**N1–Nd1–N8**	73.38(12)	72.6(4)	**N7–Nd1–N4**	139.27(12)	70.0(4)
**N1–Nd1–N9**	115.18(13)	114.7(5)	**N7–Nd1–N5**	79.04(12)	121.4(5)
**N2–Nd1–N1**	79.40(14)	78.3(5)	**N7–Nd1–N8**	68.31(11)	139.3(5)
**N2–Nd1–N3**	80.18(14)	81.1(5)	**N7–Nd1–N9**	71.37(11)	80.0(5)
**N2–Nd1–N4**	72.86(13)	73.6(5)	**N8–Nd1–N5**	133.77(11)	69.2(5)
**N2–Nd1–N5**	125.03(13)	124.4(5)	**N8–Nd1–N9**	60.35(11)	71.6(5)
**N2–Nd1–N6**	155.11(13)	153.7(5)	**N9–Nd1–N5**	137.23(11)	60.9(4)
**N2–Nd1–N7**	138.26(13)	138.1(5)	**C1–N1–Nd1**	159.2(4)	160.9(14)
**N2–Nd1–N8**	100.91(13)	99.6(5)	**N1–C1–S1**	177.9(4)	—
**N2–Nd1–N9**	68.65(13)	68.0(5)	**N2–C2–S2**	179.4(5)	—
**N3–Nd1–N4**	84.56(12)	84.9(5)	**N3–C3–S3**	177.4(6)	—
**N3–Nd1–N5**	68.93(12)	67.8(5)	**N1–C1–Se1**	—	177.2(15)
**N3–Nd1–N6**	123.70(12)	124.2(5)	**N2–C2–Se2**	—	179(2)
**N3–Nd1–N7**	78.68(13)	78.3(5)	**N3–C3–Se3**	—	174(2)
**N3–Nd1–N8**	130.86(12)	131.4(5)	**C2–N2–Nd1**	159.1(4)	159.4(16)
**N3–Nd1–N9**	75.39(12)	75.3(4)	**C3–N3–Nd1**	164.3(4)	163.0(18)
**N4–Nd1–N5**	60.28(12)	59.3(5)			
**N4–Nd1–N8**	143.35(11)	142.4(5)			
**N4–Nd1–N9**	138.92(12)	138.8(5)			

Table S3. Results of Continuous Shape Measure (CSM) analyses for the first coordination sphere of Nd(III) ion of **1-S** and **1-Se**.

Compound	Nd^{III} center (CSM parameters)				
	$S_{JCSAPR\text{-}9}$	$S_{CSAPR\text{-}9}$	$S_{JTCTPR\text{-}9}$	$S_{TCTPR\text{-}9}$	$S_{MFF\text{-}9}$
**Ideal JCSAPR-9**	0	1.133	2.359	2.314	2.135
**Ideal CSAPR-9**	1.133	0	2.924	1.169	0.817
**Ideal JTCTPR-9**	2.359	2.924	0	2.625	3.805
**Ideal TCTPR-9**	2.314	1.169	2.625	0	2.045
**Ideal MFF-9**	2.135	0.817	3.805	2.045	0
**1-S**	2.546	1.715	4.019	1.878	1.502
**1-Se**	2.55	1.687	3.924	1.878	1.385

*CSM parameters[5]: $S_{JCSAPR\text{-}9}$ - the parameter related to the capped square antiprism geometry (C_{4v} symmetry), $S_{CSAPR\text{-}9}$ - the parameter related to the spherical capped square antiprism geometry (C_{4v} symmetry), $S_{JTCTPR\text{-}9}$ - the parameter related to the tricapped trigonal prism geometry (D_{3h} symmetry), $S_{TCTPR\text{-}9}$ - the parameter related to the spherical tricapped trigonal prism geometry (D_{3h} symmetry), $S_{MFF\text{-}9}$ - the parameter related to the muffin geometry (C_s symmetry), $S = 0$ for the ideal geometry and increases with the increasing distortion from the ideal polyhedron.

Thermogravimetric analysis

The partially occupied ethanol molecules are removed from the crystal structure by 50°C in both assemblies as indicated in the TGA patterns. **1-S** shows higher stability until 250°C followed by the decomposition of three phen and three thiocyanate ligands, which account for 74% of the weight loss. On the other hand, **1-Se** begins to decompose above 180°C due to the less stable selenocyanate which decomposes to selenium and free cyanide. A further increase in temperature leads to the elimination of three cyanide and three phen, which corresponds to 58% of mass loss. These results are in agreement with those of the powder diffraction and single-crystal studies.

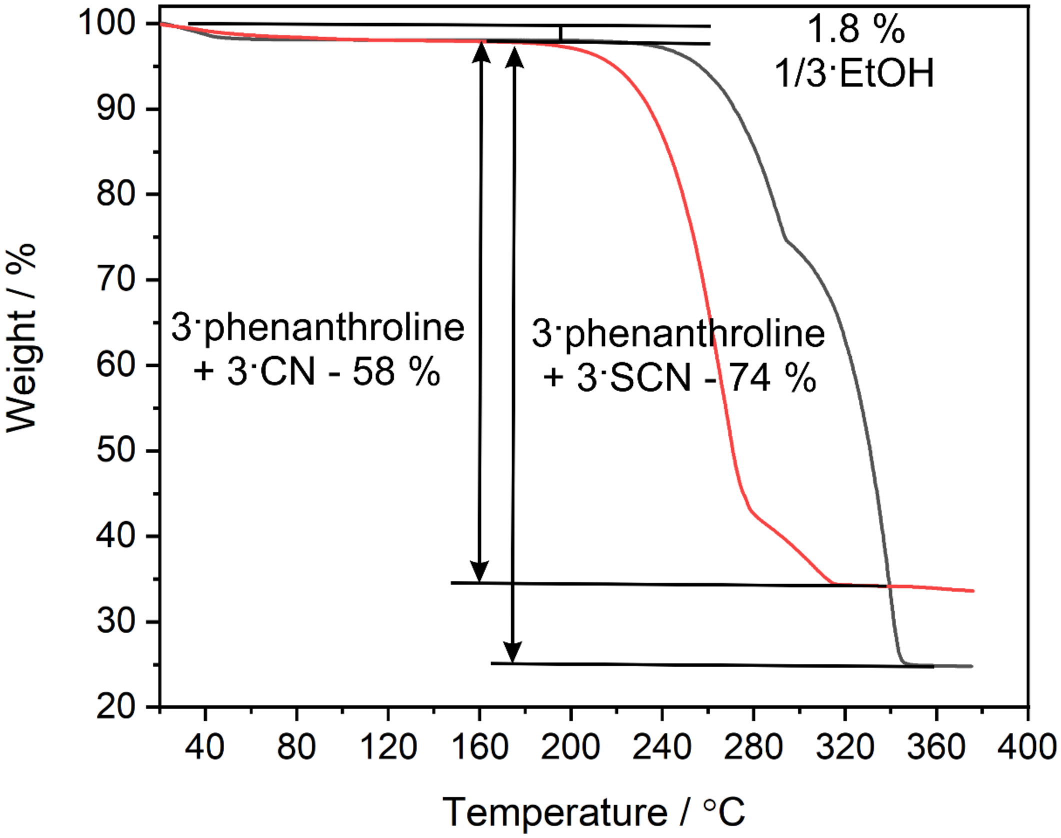

Figure S2. Thermogravimetric plots for **1-S** and **1-Se** with indicated weight losses.

Powder X-ray diffraction (PXRD) studies

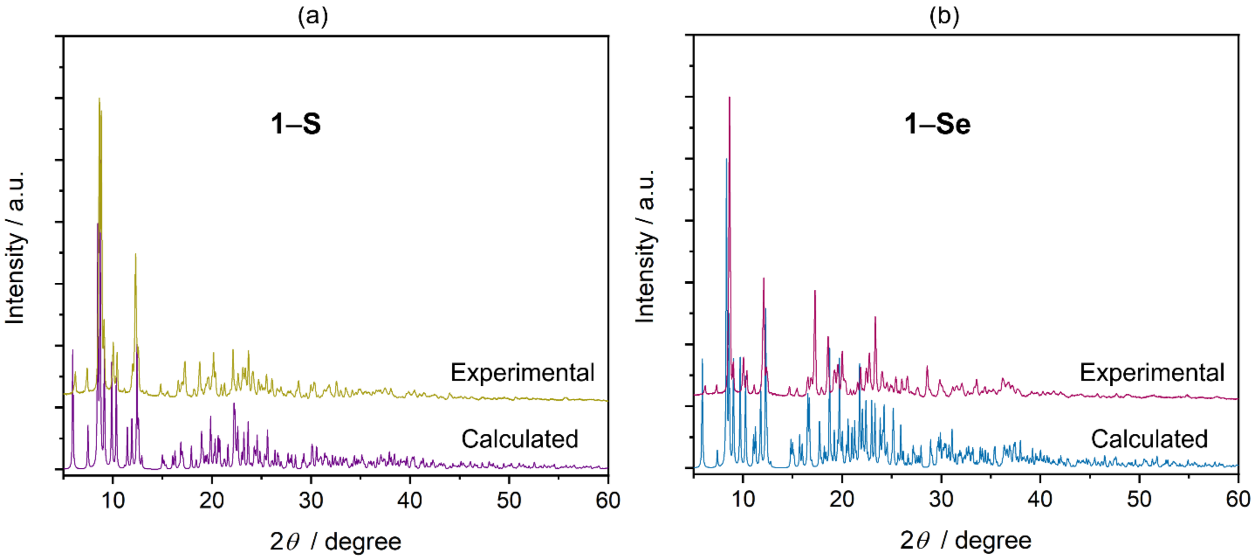

Figure S3. (a, b) Experimental and calculated powder X-ray diffraction patterns of **1**-**S** and **1**-**Se**. The calculated PXRD patterns were simulated based on the structures determined in the SXRD experiments at 300 K.

Vibrational spectroscopy

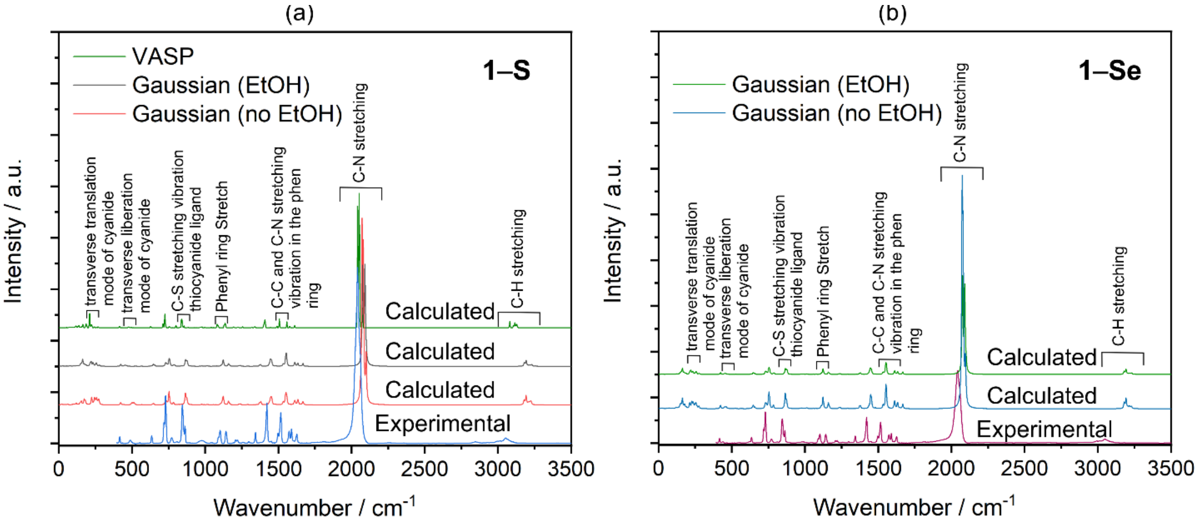

Figure S4. (a, b) Infrared (IR) absorption spectra in KBr matrix with assignment for **1**-**S** and **1**-**Se** compared with calculated IR spectra. DFT calculations were performed using Gaussian 16 for two models with and without ethanol. Panel (a) is supplemented by IR spectra determined from first-principal phonon calculation using VASP.

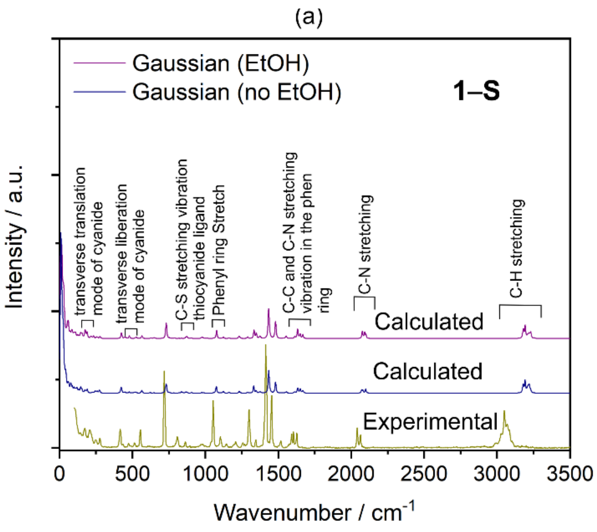

Figure S5. (a, b) Experimental Raman scattering spectra for single crystals with assignment for **1-S** and **1-Se** compared with calculated Raman spectra. DFT calculations were performed using Gaussian 16 for two models with and without ethanol in the input.

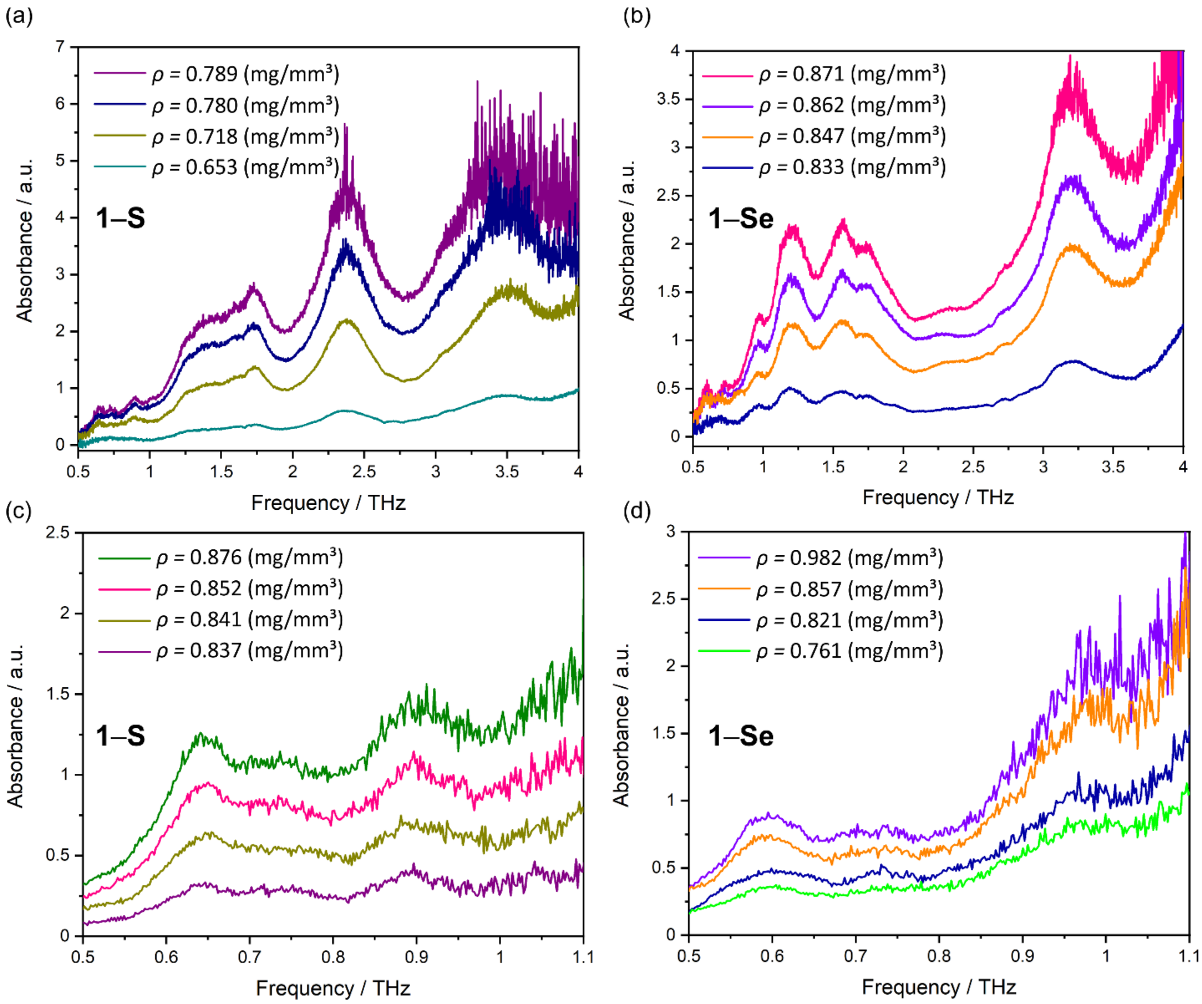

Figure S6. THz-TDS spectra of (a, c) **1-S** and (b, d) **1-Se** measured with two different measurement modules for different fill rates (ρ).

Table S4. List of IR and Raman active vibrational modes for **1-S** and **1-Se** without ethanol solvent, including relevant assignment.

Mode #	f (cm^{-1})	IR (KM/Mole)	Raman activity (Å^4/amu)	Mode#	f (cm^{-1})	IR (KM/Mole)	Raman activity (Å^4/amu)	Assignment
	**1-S** without EtOH				**1-Se** without EtOH			
**1**	7.4249	466.9135	16.53137	**1**	7.3847	454.9772	3.9382482863	
**2**	9.7835	462.1234	16.79887	**2**	12.1978	78.03622	15.2320949205	
**3**	15.6492	462.2074	6.162452	**3**	13.552	416.9262	4.7206835380	
**4**	17.9413	107.3768	7.09294	**4**	15.6588	195.74	6.2201452692	
**5**	21.1039	594.0268	3.382326	**5**	19.9346	262.9633	4.4750779374	
**6**	25.6796	221.2845	9.050351	**6**	23.9066	300.1068	5.5863735052	
**7**	33.9624	85.32666	1.568453	**7**	27.1271	171.2257	3.0406012640	
**8**	38.6337	189.3414	0.543075	**8**	32.8149	148.185	1.3415765093	
**9**	41.2238	10.28709	1.928389	**9**	34.6902	82.2255	1.5812744187	
**10**	44.8882	205.2902	1.418854	**10**	38.1421	3.409732	0.7546622257	
**11**	55.6678	2.809243	0.823534	**11**	50.379	71.48274	2.4504278637	
**12**	58.4369	74.15321	0.38073	**12**	55.1518	123.2875	1.4369017055	
**13**	60.8209	219.8006	0.495854	**13**	60.0222	93.02492	0.4610260628	
**14**	63.9928	107.0775	0.824484	**14**	69.6821	228.9999	1.5745973789	
**15**	75.8287	20.26562	1.197572	**15**	76.7394	80.51118	0.4831503758	
**16**	79.4608	17.38629	0.690252	**16**	80.8846	27.83739	1.0888683932	
**17**	86.7235	24.76712	0.198563	**17**	87.0884	6.917098	0.6153951062	
**18**	87.6824	19.86909	0.759092	**18**	89.9065	54.14805	0.5295425442	
**19**	97.6339	213.1298	0.769898	**19**	93.4713	70.81968	0.4156185566	
**20**	103.6875	8.037469	0.343079	**20**	96.1063	34.76069	0.2172053487	
**21**	108.4964	86.02309	0.420631	**21**	104.0772	34.12621	0.8894711951	
**22**	113.4303	110.59	0.306284	**22**	105.3774	8.733884	0.7257534061	
**23**	118.5026	87.51906	0.43156	**23**	116.9033	116.1397	0.0770180125	
**24**	119.1857	338.9821	0.209063	**24**	118.7883	156.6429	0.2937461598	
**25**	121.9015	56.38104	0.300364	**25**	126.4428	46.1465	0.1620397736	
**26**	131.3398	46.37902	0.521183	**26**	133.0581	134.411	0.4719722177	
**27**	143.1833	149.6059	0.49312	**27**	142.771	107.6574	0.9087139621	
**28**	145.7013	380.6459	0.725948	**28**	147.0104	411.9291	0.2947927050	Movement of phen ligand perpendicular to the aromatic ring
**29**	152.5344	155.3029	0.456788	**29**	151.8817	518.7866	0.9250059937	
**30**	154.2134	585.8627	0.175142	**30**	162.3673	1534.441	0.1076032368	

**31**	173.055 5	1004.176	0.59161	**31**	174.1478	215.5928	1.3200130957	
**32**	180.606 5	640.0013	0.273035	**32**	178.4827	428.467	0.8521814517	
**33**	189.268 2	69.83989	0.824176	**33**	188.9784	181.6692	1.7580235258	
**34**	224.000 1	1094.439	0.191808	**34**	212.3844	649.3653	0.2776395142	Transverse translation mode of cyanide
**35**	235.506 1	90.96946	0.01826	**35**	226.1299	668.5882	0.3524575287	
**36**	236.959 4	7.225919	0.090079	**36**	234.5683	449.7829	0.5738620946	
**37**	243.772 6	825.4775	0.395265	**37**	238.6361	225.9486	0.1674487832	
**38**	250.023 3	279.6945	0.058383	**38**	248.4767	278.539	0.0970167824	
**39**	251.602 6	12.25823	0.159744	**39**	253.485	170.7122	0.1703220091	
**40**	257.385 6	482.5596	0.136873	**40**	254.8022	226.5623	0.2057698040	
**41**	259.009 3	343.7536	0.219818	**41**	256.9626	126.5335	0.0639895909	
**42**	269.758 1	380.5246	0.159135	**42**	262.9795	120.65	0.1107497968	Movement of phen ligand along aromatic ring (ring stretch)
**43**	272.477 6	210.454	0.145779	**43**	275.1005	76.03914	0.2144855214	
**44**	273.510 8	96.78465	0.281089	**44**	275.4573	108.8557	0.0673308124	
**45**	275.787 6	160.3758	0.221647	**45**	276.2837	19.67815	0.2578155243	
**46**	417.955 1	3.393261	0.062525	**46**	420.7954	39.044	0.1301512948	
**47**	419.814 2	21.55038	0.218134	**47**	421.5903	57.4776	0.3645266317	
**48**	421.452 7	35.11821	0.233909	**48**	422.1799	70.76462	0.4642178063	
**49**	421.581 2	83.86341	0.549873	**49**	423.7286	45.46683	0.1659391694	
**50**	424.080 1	46.01428	0.355529	**50**	424.5498	7.818114	0.3404444617	
**51**	425.05	17.29975	0.252708	**51**	426.4382	5.646774	0.1688734891	
**52**	442.436 7	14.05731	0.135231	**52**	443.5971	10.9943	0.0747767675	
**53**	448.684 2	3.820603	0.091636	**53**	444.3116	2.872327	0.1880735695	
**54**	449.936 8	6.711109	0.111385	**54**	446.5061	12.71229	0.0284329389	

**55**	474.3522	3.104205	0.086564	**55**	449.1756	30.35819	0.0753042535	
**56**	476.5471	4.078578	0.148591	**56**	450.7264	5.638998	0.1053586873	
**57**	477.0303	3.57434	0.073215	**57**	453.067	48.25665	0.0122488099	
**58**	494.8747	5.292328	0.011156	**58**	454.4659	26.71648	0.0809301109	Transverse liberation mode of cyanide
**59**	495.9401	59.23764	0.021044	**59**	459.6967	44.43736	0.0746112222	
**60**	500.4711	25.14302	0.024852	**60**	464.0461	65.02158	0.0397568033	
**61**	501.3931	86.50762	0.006792	**61**	475.6187	8.658732	0.1958009677	
**62**	509.5946	65.36543	0.048787	**62**	476.9125	9.969468	0.3032278944	
**63**	512.4422	76.08736	0.087673	**63**	478.9735	2.290491	0.2125199477	
**64**	515.8621	0.128375	0.067252	**64**	516.6626	3.369652	0.1103476474	
**65**	516.736	52.05619	0.081718	**65**	521.6282	10.18174	0.0978083914	Various motions related to the perpendicular and parallel stretching of phen aromatic rings. Some motions also related to the slant movement of rings
**66**	521.5709	7.670228	0.053003	**66**	523.0544	5.848474	0.0819395398	
**67**	523.4271	3.864957	0.036599	**67**	524.3397	5.694143	0.0653227884	
**68**	524.5903	2.962825	0.086073	**68**	525.5708	3.373262	0.0435160129	
**69**	525.3042	1.906967	0.055293	**69**	526.507	9.697933	0.1719935599	
**70**	559.9303	0.751668	0.049176	**70**	559.1528	2.736878	0.0334136046	
**71**	561.6104	0.961103	0.054507	**71**	562.6026	0.382203	0.1095201497	
**72**	563.046	2.037048	0.239026	**72**	563.436	1.210762	0.3072984777	
**73**	563.6739	0.036095	0.277017	**73**	564.1764	6.775612	0.1397759712	
**74**	564.1134	16.46851	0.060884	**74**	565.0924	7.19739	0.1298242855	
**75**	565.8075	1.323436	0.05755	**75**	567.3454	2.980031	0.0933231189	
**76**	618.9055	1.229231	0.09056	**76**	620.5252	0.480251	0.0469522282	
**77**	620.7227	0.712114	0.069427	**77**	622.9342	4.32732	0.0693671358	
**78**	623.3492	2.474217	0.021592	**78**	624.6482	4.183882	0.0607108496	
**79**	638.9163	23.06161	0.015303	**79**	636.2264	23.16225	0.0687101390	

**80**	645.0785	40.2373	0.047258	**80**	642.2029	34.61417	0.0115918647	
**81**	647.6287	44.71734	0.033342	**81**	645.5904	2.941421	0.1430987516	
**82**	723.9932	47.51836	0.701754	**82**	645.9121	56.34586	0.1015327556	
**83**	729.204	97.59855	0.90287	**83**	647.7679	65.77227	0.0311509451	
**84**	731.9271	71.64025	1.473149	**84**	657.6545	47.47936	0.0876390790	
**85**	735.9896	21.29317	0.007797	**85**	726.6234	62.96303	0.8829484827	
**86**	736.7835	31.81627	0.013298	**86**	729.3674	96.75241	1.2173721583	
**87**	738.1455	34.37826	0.018494	**87**	731.899	44.80243	2.5241081040	
**88**	752.7637	226.9058	0.040834	**88**	736.246	36.87551	0.0181639796	
**89**	752.8044	334.648	0.039486	**89**	736.7352	26.47432	0.0346083157	
**90**	755.7492	55.04134	0.017925	**90**	737.2089	36.83384	0.0301430531	
**91**	781.9715	21.45629	0.004991	**91**	751.5352	202.947	0.0750516303	
**92**	782.6904	79.1676	0.033384	**92**	754.0346	210.2967	0.0408700505	
**93**	783.8418	5.148074	0.011764	**93**	755.6542	235.3539	0.0210214462	
**94**	816.4251	2.087968	0.027514	**94**	781.5068	21.47313	0.0338141904	
**95**	818.5273	7.513561	0.030188	**95**	783.3687	35.0148	0.0792409098	
**96**	819.6307	7.094106	0.030172	**96**	784.5092	62.29438	0.0758853025	
**97**	835.2359	7.891522	0.083497	**97**	814.5299	5.910165	0.0267021669	
**98**	836.0269	3.957298	0.273055	**98**	817.3071	2.44936	0.1860799625	C–S stretching vibration from thiocyanate ligand
**99**	843.8778	10.76962	0.135666	**99**	818.9019	2.595611	0.0206638772	
**100**	861.771	13.75412	0.030579	**100**	861.8109	4.673989	0.0748959858	
**101**	862.9746	49.25502	0.075057	**101**	863.287	64.56978	0.0761192105	
**102**	863.887	196.9709	0.049735	**102**	863.4618	11.2512	0.1058963665	
**103**	864.124	66.18541	0.049491	**103**	864.6956	202.5745	0.1510994807	
**104**	865.5799	50.92684	0.06557	**104**	865.195	79.2291	0.0463353675	
**105**	866.6161	75.13333	0.024975	**105**	866.0929	82.26582	0.0955487597	

**106**	870.5185	50.04674	0.077741	**106**	872.1529	43.25176	0.2135150871	
**107**	873.1257	71.05725	0.132314	**107**	873.3524	63.55291	0.2564336884	
**108**	875.0379	117.2923	0.055948	**108**	874.4471	94.38541	0.0681922237	
**109**	898.1086	0.943925	0.029533	**109**	900.8644	0.097425	0.0395905159	
**110**	903.1393	1.913997	0.070155	**110**	903.4979	1.997573	0.0652756539	
**111**	905.1613	2.596833	0.113434	**111**	904.5079	3.614905	0.1130485673	
**112**	967.7616	4.473925	0.023523	**112**	962.8913	13.49422	0.1281394490	
**113**	973.4537	13.72893	0.15034	**113**	974.8209	16.1545	0.3646205604	C–H motion of the phen ligand in various directions above the aromatic ring
**114**	976.7662	6.737038	0.021012	**114**	978.1832	1.38053	0.1837740639	
**115**	983.623	0.551997	0.012406	**115**	980.5889	3.112302	0.0449037592	
**116**	984.0917	0.362418	0.018204	**116**	982.7941	2.664485	0.0705887449	
**117**	984.2792	2.416873	0.030801	**117**	985.6859	2.552247	0.0508044943	
**118**	991.4072	1.276807	0.021591	**118**	989.4026	3.216831	0.0148402634	
**119**	992.9519	2.459645	0.01114	**119**	991.0183	0.157399	0.0781764503	
**120**	993.4476	3.554706	0.008737	**120**	994.0734	6.761409	0.0395701085	
**121**	1004.839	2.364642	0.003189	**121**	1003.625	2.705378	0.0148903155	
**122**	1009.341	7.181651	0.017164	**122**	1009.096	7.75506	0.0291096292	
**123**	1010.139	0.095969	0.003535	**123**	1010.228	10.35982	0.0082383498	
**124**	1014.374	6.78144	0.004613	**124**	1012.601	2.775163	0.0389975997	
**125**	1019.231	7.272443	0.013205	**125**	1014.781	7.551222	0.0100935065	
**126**	1023.147	1.741748	0.011369	**126**	1020.074	7.753729	0.0376575351	
**127**	1054.49	2.669839	0.007431	**127**	1055.095	2.262219	0.0183947240	Phenyl–ring breathing vibration of phenanthroline ligands
**128**	1054.659	0.687306	0.027959	**128**	1056.114	4.122312	0.0463231708	
**129**	1056.388	6.935453	0.025545	**129**	1056.202	3.514227	0.0404872668	
**130**	1071.187	3.252039	0.349177	**130**	1073.756	3.216394	0.6853416142	

**131**	1074.541	1.687401	0.201605	**131**	1075.185	1.53315	0.7208182882	
**132**	1074.867	1.101581	1.428968	**132**	1076.764	2.984367	1.1151782326	
**133**	1104.115	8.052394	0.006382	**133**	1106.861	9.242364	0.0148935388	
**134**	1107.543	17.18527	0.013291	**134**	1108.654	15.24649	0.0415003675	
**135**	1108.388	12.89801	0.024584	**135**	1110.075	20.68632	0.0108200445	
**136**	1117.418	64.08994	0.075986	**136**	1120.527	68.87586	0.0577342813	Phenyl ring stretch
**137**	1121.612	118.7186	0.171205	**137**	1123.002	207.097	0.1165231201	
**138**	1123.68	138.0285	0.3	**138**	1123.399	6.860182	0.3057141313	
**139**	1157.224	29.08628	0.04869	**139**	1158.4	46.97839	0.0391957345	
**140**	1157.792	43.3782	0.044898	**140**	1158.98	37.84623	0.0557387667	
**141**	1159.918	39.37849	0.050406	**141**	1159.67	41.55006	0.0492542923	
**142**	1165.215	7.31243	0.033919	**142**	1166.701	15.87038	0.0532300013	
**143**	1165.791	9.277187	0.035001	**143**	1166.814	10.15732	0.0231970276	
**144**	1167.111	14.24903	0.041493	**144**	1167.048	5.602362	0.0255641928	
**145**	1227.338	1.548839	0.016728	**145**	1227.578	2.573527	0.0972407518	
**146**	1229.142	2.400502	0.063257	**146**	1228.625	5.915138	0.3186859367	
**147**	1229.838	8.662999	0.070463	**147**	1230.521	4.000353	0.1715311990	
**148**	1230.553	0.550808	0.080467	**148**	1231.831	0.357217	0.0454616296	
**149**	1231.558	1.085492	0.1435	**149**	1232.539	2.425606	0.1625926607	
**150**	1234.302	15.124	0.274912	**150**	1234.243	10.24886	0.1576260196	
**151**	1240.236	6.375389	0.013515	**151**	1240.754	7.137325	0.0269014696	
**152**	1241.726	8.858611	0.019692	**152**	1241.482	7.70031	0.0386964059	
**153**	1242.543	7.026877	0.008756	**153**	1242.391	6.858514	0.0183842360	
**154**	1284.639	5.186433	0.071235	**154**	1287.945	1.177046	0.1688335009	

**155**	1288.832	7.28895	0.121328	**155**	1289.502	6.289286	0.3132063169	
**156**	1293.635	6.43942	0.057402	**156**	1292.191	7.528718	0.1547241141	
**157**	1330.263	1.83296	0.36681	**157**	1331.87	3.001027	0.9015144130	
**158**	1330.667	4.384039	0.76213	**158**	1332.607	1.215134	1.0285905113	
**159**	1331.213	0.566997	0.941911	**159**	1333.876	0.940018	0.7161142267	
**160**	1347.176	0.169387	0.212968	**160**	1347.817	4.194468	0.8711687967	
**161**	1348.238	2.446182	0.192141	**161**	1349.308	0.41511	0.1186248838	C–N movement of phen ligand
**162**	1348.461	2.793695	0.252876	**162**	1349.604	1.175	0.6458464500	
**163**	1369.492	28.74044	0.107992	**163**	1372.254	21.80942	0.1027744452	
**164**	1372.831	26.02843	0.256846	**164**	1373.065	20.14361	0.5562519439	
**165**	1379.575	75.85224	0.187984	**165**	1378.414	48.14203	0.3030796499	
**166**	1432.944	4.540519	1.846455	**166**	1432.431	7.910669	2.6119246523	
**167**	1433.4	4.124939	2.236525	**167**	1432.775	1.974129	4.0416527017	
**168**	1435.061	10.71142	2.644636	**168**	1435.527	6.260081	3.1552832771	
**169**	1436.282	1.251025	0.228554	**169**	1437.262	3.361919	0.1168582654	
**170**	1437.495	8.445339	0.126887	**170**	1437.803	9.788128	0.1203703676	
**171**	1438.667	8.778706	0.165541	**171**	1437.992	7.899219	0.2100029607	
**172**	1444.091	93.47642	0.074302	**172**	1446.429	91.57533	0.1372743785	
**173**	1449.125	116.3782	0.06843	**173**	1448.415	127.0273	0.0537543850	
**174**	1454.258	115.4868	0.0242	**174**	1453.33	121.0989	0.0568029988	
**175**	1478.195	1.722933	0.626054	**175**	1479.754	2.243062	1.2526051078	
**176**	1479.949	5.759757	1.524519	**176**	1480.096	8.170188	2.2382781907	
**177**	1481.971	5.113365	1.444814	**177**	1481.951	4.56454	2.2491215966	
**178**	1531.039	10.36149	0.019714	**178**	1532.454	23.58149	0.0274042257	C–C and C–N bond

**179**	1532.861	20.71708	0.021935	**179**	1532.697	13.39615	0.0280994126	stretching vibrations within phenanthroline rings
**180**	1532.904	29.12624	0.016266	**180**	1533.373	19.03205	0.0356287703	
**181**	1546.956	113.0249	0.095578	**181**	1550.392	151.7013	0.2253979602	
**182**	1551.756	175.9568	0.204637	**182**	1552.917	197.4658	0.4732027071	
**183**	1556.854	125.1038	0.256145	**183**	1555.166	119.9051	0.2257791769	
**184**	1607.225	24.66454	0.052129	**184**	1609.947	62.38138	0.1126650537	
**185**	1610.859	51.44546	0.212071	**185**	1611.023	33.61916	0.3392268926	
**186**	1614.394	40.54182	0.174215	**186**	1613.451	39.31512	0.2093533916	
**187**	1630.393	17.57016	0.510327	**187**	1630.892	23.94678	1.3778508401	
**188**	1631.98	47.95427	0.632001	**188**	1631.133	26.25524	1.2398445798	
**189**	1634.71	57.00555	0.64656	**189**	1632.433	51.83889	0.6274663532	
**190**	1647.401	0.910291	0.194523	**190**	1647.904	0.947053	0.2857339918	
**191**	1648.974	4.966865	0.53124	**191**	1648.683	5.56784	0.5911366027	
**192**	1650.519	3.181089	0.646535	**192**	1649.191	3.356125	0.6945858784	
**193**	1665.273	19.56185	0.175662	**193**	1666.406	25.5185	0.4885387212	
**194**	1666.44	32.44373	0.398392	**194**	1667.934	28.31173	0.4552427565	
**195**	1670.036	23.82503	0.363547	**195**	1668.021	20.14118	0.5327519546	
**196**	2070.869	2618.087	1.050787	**196**	2072.794	2363.177	1.9820562789	Cyanide bond stretching
**197**	2079.316	2153.297	0.860805	**197**	2079.514	1714.718	2.0926683647	
**198**	2098.666	722.2394	1.423141	**198**	2095.448	495.8099	1.8349106498	
**199**	3170.553	0.260335	0.156264	**199**	3172.061	0.192297	0.1504067885	C–H bond stretching of phenanthroline rings
**200**	3171.423	0.075853	0.122452	**200**	3172.456	0.095822	0.1522659827	
**201**	3173.237	0.207564	0.161895	**201**	3172.938	0.195262	0.1744519909	
**202**	3177.046	9.020906	0.475221	**202**	3177.896	8.648286	0.6228980398	
**203**	3177.752	6.337461	0.660337	**203**	3178.885	10.7375	0.7487846630	

**204**	3178.051	9.049435	0.463216	**204**	3179.006	7.334504	0.6608305284	
**205**	3178.378	13.58618	0.83132	**205**	3180.481	8.624953	0.7316721956	
**206**	3178.457	6.364045	0.745106	**206**	3180.712	7.758771	0.7327134984	
**207**	3180.876	5.339053	0.610052	**207**	3181.253	7.284554	0.6994288949	
**208**	3189.897	18.86508	1.12479	**208**	3191.347	22.30582	1.3640618779	
**209**	3190.667	29.5225	1.894567	**209**	3191.572	31.51022	2.0715265363	
**210**	3191.533	32.6641	1.054431	**210**	3192.084	27.71858	2.2106365154	
**211**	3192.622	18.91071	1.600654	**211**	3203.641	8.992094	0.5886861505	
**212**	3203.528	7.111364	0.355898	**212**	3210.016	1.012258	0.4726409320	
**213**	3205.188	3.146025	0.689402	**213**	3211.655	4.683946	0.5776289500	
**214**	3208.001	5.410556	0.57139	**214**	3213.047	4.434835	0.8662072767	
**215**	3210.66	7.786673	0.596072	**215**	3213.201	1.915114	0.2801202484	
**216**	3213.011	13.74668	1.585271	**216**	3214.111	0.520192	0.1965886190	
**217**	3217.779	5.50483	1.155892	**217**	3216.828	11.9072	1.4507493714	
**218**	3217.843	0.420035	0.447758	**218**	3224.371	4.50995	0.9648406790	
**219**	3220.014	7.974919	1.222246	**219**	3225.843	3.704038	1.0016476142	
**220**	3221.632	13.37616	0.734614	**220**	3229.346	10.18429	0.8354897004	
**221**	3226.234	26.6903	0.735192	**221**	3230.668	2.963151	0.5549808622	
**222**	3231.127	1.540136	0.674748	**222**	3238.518	1.341495	0.2135558855	

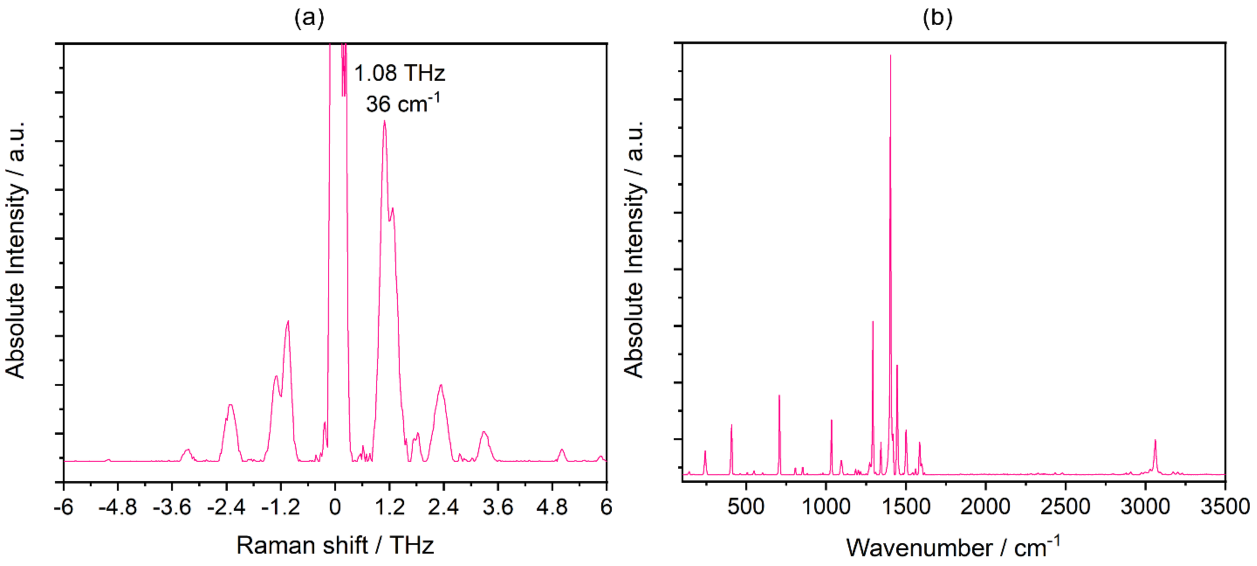

Figure S7. Experimental Raman scattering spectra for single crystals of phenanthroline in (a) the low-frequency region of -6–6 THz (-200–200 cm^{-1}) with Stokes and anti-Stokes Raman scattering signals and (b) the high-frequency region of 100–3500 cm^{-1}.

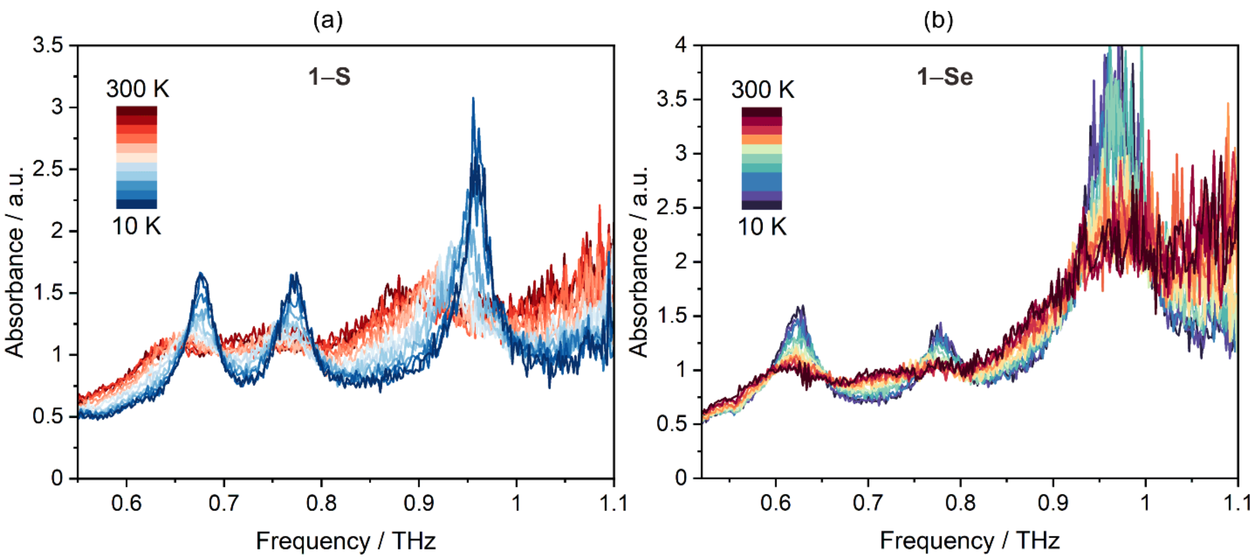

Figure S8. Temperature-dependent THz-TDS spectra of (a) **1-S** and (b) **1-Se** determined in the range of 300–10 K.

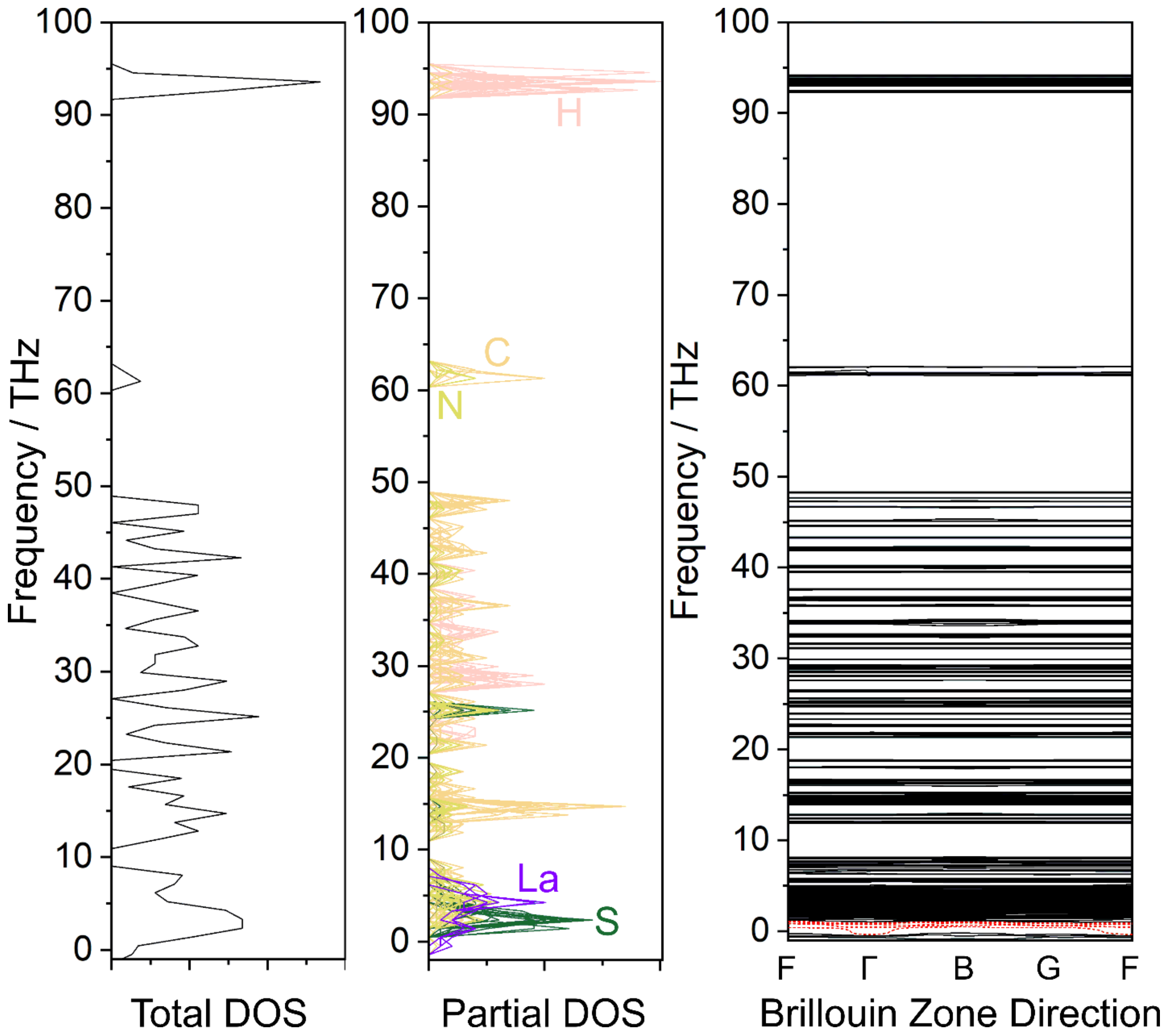

Figure S9. Total DOS of the **1**-**S** (black line) and partial phonon DOS of La (magenta line), S (dark green line), C (light orange line), N (light green line), and H (light pink line). The band structure of **1**-**S**. The dotted red-lines close to zero correspond to the vibrational band structure characterized through THz-TDS.

Table S5. Calculated optical phonon modes obtained through first-principles calculations of **1**-**S**.

Frequency (THz)	Frequency (cm^{-1})	IR (a.u.)	Irreducible Representation	Frequency (THz)	Frequency (cm^{-1})	IR (a.u.)	Irreducible Representation
**-0.682**	-22.73311	0.0000089	A(RI)	26.359	878.62455	0.0000720	A(RI)
**-0.499**	-16.63317	0.0000007	A(RI)	26.38	879.32454	0.0001070	A(RI)
**-0.458**	-15.26651	0.0002081	A(RI)	26.474	882.45784	0.0002071	A(RI)
**-0.342**	-11.39989	0.0000321	A(RI)	26.484	882.79117	0.0000130	A(RI)
**0.445**	14.83319	0.0005346	A(RI)	26.56	885.32448	0.0000039	A(RI)
**0.753**	25.09975	0.0001690	A(RI)	26.575	885.82448	0.0000136	A(RI)
**0.844**	28.13305	0.0000041	A(RI)	27.613	920.42413	0.0000696	A(RI)
**0.936**	31.19969	0.0000642	A(RI)	27.633	921.09079	0.0010616	A(RI)
**1.071**	35.69964	0.0002163	A(RI)	28.008	933.59066	0.0000069	A(RI)
**1.302**	43.39957	0.0000814	A(RI)	28.032	934.39066	0.0001138	A(RI)
**1.453**	48.43285	0.0003277	A(RI)	28.095	936.49064	0.0002707	A(RI)
**1.508**	50.26616	0.0001514	A(RI)	28.116	937.19063	0.0000189	A(RI)
**1.564**	52.13281	0.0000155	A(RI)	28.132	937.72396	0.0002543	A(RI)
**1.583**	52.76614	0.0011000	A(RI)	28.148	938.25728	0.0001550	A(RI)
**1.676**	55.86611	0.0000248	A(RI)	28.444	948.12385	0.0000000	A(RI)
**1.714**	57.13276	0.0000203	A(RI)	28.469	948.95718	0.0002094	A(RI)
**1.808**	60.26606	0.0000104	A(RI)	28.593	953.09047	0.0001762	A(RI)
**1.897**	63.2327	0.0000904	A(RI)	28.623	954.09046	0.0007153	A(RI)
**1.992**	66.39934	0.0003814	A(RI)	28.834	961.12372	0.0000462	A(RI)
**2.004**	66.79933	0.0001005	A(RI)	28.853	961.75705	0.0000118	A(RI)
**2.077**	69.23264	0.0000247	A(RI)	28.859	961.95705	0.0000100	A(RI)
**2.16**	71.99928	0.0002613	A(RI)	28.871	962.35704	0.0000924	A(RI)
**2.23**	74.33259	0.0000234	A(RI)	28.913	963.75703	0.0000208	A(RI)
**2.283**	76.09924	0.0003414	A(RI)	28.942	964.72369	0.0010427	A(RI)
**2.309**	76.9659	0.0002406	A(RI)	29.011	967.02366	0.0030085	A(RI)
**2.369**	78.96588	0.0000635	A(RI)	29.035	967.82366	0.0018610	A(RI)
**2.438**	81.26585	0.0005202	A(RI)	29.165	972.15695	0.0000555	A(RI)
**2.449**	81.63252	0.0000387	A(RI)	29.194	973.1236	0.0000046	A(RI)
**2.472**	82.39918	0.0000166	A(RI)	29.27	975.65691	0.0013443	A(RI)
**2.517**	83.89916	0.0007048	A(RI)	29.298	976.59023	0.0000203	A(RI)
**2.585**	86.16581	0.0004946	A(RI)	29.31	976.99023	0.0019104	A(RI)
**2.656**	88.53245	0.0002751	A(RI)	29.322	977.39023	0.0005182	A(RI)
**2.695**	89.83244	0.0000112	A(RI)	29.91	996.99003	0.0006134	A(RI)
**2.753**	91.76575	0.0000170	A(RI)	29.919	997.29003	0.0010469	A(RI)
**2.781**	92.69907	0.0003807	A(RI)	29.93	997.65669	0.0007933	A(RI)
**2.825**	94.16573	0.0000094	A(RI)	29.938	997.92335	0.0002648	A(RI)
**2.899**	96.63237	0.0004937	A(RI)	31.083	1036.08964	0.0000014	A(RI)
**2.957**	98.56568	0.0002065	A(RI)	31.108	1036.92296	0.0000425	A(RI)
**3.013**	100.43233	0.0002026	A(RI)	31.157	1038.55628	0.0000449	A(RI)
**3.11**	103.66563	0.0000474	A(RI)	31.163	1038.75628	0.0004187	A(RI)
**3.118**	103.93229	0.0002323	A(RI)	31.179	1039.28961	0.0021223	A(RI)
**3.207**	106.89893	0.0000097	A(RI)	31.242	1041.38959	0.0008567	A(RI)
**3.217**	107.23226	0.0003035	A(RI)	31.561	1052.02281	0.0000009	A(RI)

**3.277**	109.23224	0.0000556	A(RI)	31.568	1052.25614	0.0000028	A(RI)
**3.287**	109.56557	0.0010500	A(RI)	31.583	1052.75614	0.0000004	A(RI)
**3.369**	112.29888	0.0004986	A(RI)	31.602	1053.38947	0.0001106	A(RI)
**3.423**	114.09886	0.0006635	A(RI)	31.673	1055.75611	0.0003885	A(RI)
**3.453**	115.09885	0.0004027	A(RI)	31.725	1057.48943	0.0001499	A(RI)
**3.488**	116.2655	0.0001644	A(RI)	32.351	1078.35588	0.0018242	A(RI)
**3.57**	118.99881	0.0056500	A(RI)	32.387	1079.55587	0.0000382	A(RI)
**3.643**	121.43212	0.0000660	A(RI)	32.406	1080.1892	0.0003140	A(RI)
**3.688**	122.9321	0.0002536	A(RI)	32.413	1080.42253	0.0052762	A(RI)
**3.731**	124.36542	0.0003509	A(RI)	32.44	1081.32252	0.0029272	A(RI)
**3.819**	127.29873	0.0002858	A(RI)	32.449	1081.62252	0.0000194	A(RI)
**3.862**	128.73205	0.0004908	A(RI)	32.581	1086.02247	0.0026154	A(RI)
**3.894**	129.7987	0.0001076	A(RI)	32.609	1086.9558	0.0004614	A(RI)
**3.895**	129.83204	0.0000117	A(RI)	32.636	1087.85579	0.0014094	A(RI)
**3.934**	131.13202	0.0047700	A(RI)	32.661	1088.68911	0.0005365	A(RI)
**4.019**	133.96533	0.0004516	A(RI)	32.716	1090.52243	0.0004416	A(RI)
**4.104**	136.79863	0.0040600	A(RI)	32.739	1091.28909	0.0001967	A(RI)
**4.152**	138.39862	0.0008143	A(RI)	33.82	1127.32206	0.0002331	A(RI)
**4.206**	140.1986	0.0000647	A(RI)	33.836	1127.85539	0.0000153	A(RI)
**4.292**	143.06524	0.0007940	A(RI)	33.884	1129.45537	0.0097434	A(RI)
**4.344**	144.79855	0.0000568	A(RI)	33.893	1129.75537	0.0008351	A(RI)
**4.432**	147.73186	0.0003050	A(RI)	34.018	1133.92199	0.0187191	A(RI)
**4.462**	148.73185	0.0058000	A(RI)	34.03	1134.32199	0.0000779	A(RI)
**4.572**	152.39848	0.0007843	A(RI)	34.078	1135.92197	0.0008408	A(RI)
**4.616**	153.86513	0.0000121	A(RI)	34.106	1136.8553	0.0028335	A(RI)
**4.7**	156.6651	0.0023700	A(RI)	34.133	1137.75529	0.0029058	A(RI)
**4.766**	158.86508	0.0031700	A(RI)	34.194	1139.7886	0.0020129	A(RI)
**4.884**	162.79837	0.0045800	A(RI)	34.203	1140.0886	0.0001866	A(RI)
**4.902**	163.39837	0.0069800	A(RI)	34.213	1140.42193	0.0003600	A(RI)
**4.943**	164.76502	0.0078900	A(RI)	35.757	1191.88808	0.0040026	A(RI)
**5.071**	169.03164	0.0020400	A(RI)	35.79	1192.98807	0.0000717	A(RI)
**5.439**	181.29819	0.0013800	A(RI)	35.841	1194.68805	0.0000000	A(RI)
**5.514**	183.79816	0.0018400	A(RI)	35.86	1195.32138	0.0000316	A(RI)
**5.585**	186.16481	0.0193600	A(RI)	35.888	1196.2547	0.0000029	A(RI)
**5.649**	188.29812	0.0018000	A(RI)	35.901	1196.68803	0.0000005	A(RI)
**5.751**	191.69808	0.0017900	A(RI)	36.38	1212.65454	0.0000490	A(RI)
**5.858**	195.26471	0.0000752	A(RI)	36.404	1213.45453	0.0001675	A(RI)
**6.259**	208.63125	0.0774400	A(RI)	36.494	1216.4545	0.0000459	A(RI)
**6.312**	210.3979	0.0305600	A(RI)	36.524	1217.45449	0.0005833	A(RI)
**6.678**	222.59777	0.0008061	A(RI)	36.553	1218.42115	0.0000764	A(RI)
**6.713**	223.76443	0.0017200	A(RI)	36.559	1218.62115	0.0000582	A(RI)
**6.84**	227.99772	0.0068300	A(RI)	36.659	1221.95445	0.0002031	A(RI)
**6.997**	233.231	0.0001218	A(RI)	36.736	1224.52109	0.0000781	A(RI)
**7.198**	239.93093	0.0001245	A(RI)	36.744	1224.78775	0.0004593	A(RI)
**7.281**	242.69757	0.0033500	A(RI)	36.76	1225.32108	0.0002580	A(RI)
**7.284**	242.79757	0.0001663	A(RI)	36.781	1226.02107	0.0000962	A(RI)
**7.323**	244.09756	0.0010100	A(RI)	36.802	1226.72107	0.0022409	A(RI)

**7.417**	247.23086	0.0005295	A(RI)	37.565	1252.15415	0.0008427	A(RI)
**7.562**	252.06415	0.0000554	A(RI)	37.591	1253.0208	0.0000202	A(RI)
**7.607**	253.56413	0.0002124	A(RI)	37.614	1253.78746	0.0098188	A(RI)
**7.613**	253.76413	0.0000933	A(RI)	37.673	1255.75411	0.0000636	A(RI)
**7.669**	255.63078	0.0006787	A(RI)	37.684	1256.12077	0.0001129	A(RI)
**7.734**	257.79742	0.0006567	A(RI)	37.71	1256.98743	0.0001954	A(RI)
**7.975**	265.83068	0.0005211	A(RI)	39.475	1315.82018	0.0011049	A(RI)
**8.015**	267.164	0.0005811	A(RI)	39.491	1316.3535	0.0000359	A(RI)
**8.04**	267.99732	0.0001641	A(RI)	39.504	1316.78683	0.0000487	A(RI)
**8.064**	268.79731	0.0010500	A(RI)	39.521	1317.35349	0.0000220	A(RI)
**8.119**	270.63063	0.0011500	A(RI)	39.608	1320.25346	0.0000627	A(RI)
**8.144**	271.46395	0.0003332	A(RI)	39.628	1320.92012	0.0000813	A(RI)
**11.883**	396.09604	0.0000006	A(RI)	39.973	1332.42001	0.0006072	A(RI)
**11.957**	398.56268	0.0000001	A(RI)	40.004	1333.45333	0.0000446	A(RI)
**11.987**	399.56267	0.0000820	A(RI)	40.026	1334.18666	0.0003291	A(RI)
**12.016**	400.52933	0.0000078	A(RI)	40.041	1334.68665	0.0000656	A(RI)
**12.077**	402.56264	0.0001077	A(RI)	40.059	1335.28665	0.0005078	A(RI)
**12.097**	403.2293	0.0000350	A(RI)	40.083	1336.08664	0.0000771	A(RI)
**12.369**	412.29588	0.0002427	A(RI)	40.096	1336.51997	0.0008284	A(RI)
**12.386**	412.86254	0.0004801	A(RI)	40.117	1337.21996	0.0013387	A(RI)
**12.418**	413.92919	0.0007795	A(RI)	40.204	1340.11993	0.0007624	A(RI)
**12.429**	414.29586	0.0000323	A(RI)	40.23	1340.98659	0.0001182	A(RI)
**12.464**	415.46251	0.0003891	A(RI)	40.239	1341.28659	0.0003677	A(RI)
**12.501**	416.69583	0.0007649	A(RI)	40.282	1342.71991	0.0000300	A(RI)
**12.748**	424.92908	0.0000714	A(RI)	41.884	1396.11937	0.0001286	A(RI)
**12.77**	425.66241	0.0001640	A(RI)	41.9	1396.6527	0.0005336	A(RI)
**12.8**	426.6624	0.0000015	A(RI)	41.914	1397.11936	0.0001134	A(RI)
**12.821**	427.36239	0.0000970	A(RI)	41.921	1397.35269	0.0000532	A(RI)
**12.843**	428.09572	0.0000177	A(RI)	42	1399.986	0.0000731	A(RI)
**12.893**	429.76237	0.0004750	A(RI)	42.014	1400.45266	0.0006492	A(RI)
**13.942**	464.72869	0.0000148	A(RI)	42.016	1400.51933	0.0025249	A(RI)
**13.96**	465.32868	0.0000168	A(RI)	42.034	1401.11932	0.0062694	A(RI)
**13.988**	466.262	0.0000010	A(RI)	42.055	1401.81932	0.0094127	A(RI)
**13.998**	466.59533	0.0000008	A(RI)	42.057	1401.88598	0.0005171	A(RI)
**14.081**	469.36197	0.0000219	A(RI)	42.059	1401.95265	0.0018691	A(RI)
**14.087**	469.56197	0.0000183	A(RI)	42.07	1402.31931	0.0001440	A(RI)
**14.191**	473.0286	0.0007761	A(RI)	42.173	1405.75261	0.0161648	A(RI)
**14.218**	473.92859	0.0004721	A(RI)	42.191	1406.3526	0.0022162	A(RI)
**14.259**	475.29525	0.0009011	A(RI)	42.217	1407.21926	0.0119535	A(RI)
**14.303**	476.7619	0.0015500	A(RI)	42.282	1409.38591	0.0000189	A(RI)
**14.334**	477.79522	0.0006202	A(RI)	42.307	1410.21923	0.0000892	A(RI)
**14.362**	478.72855	0.0000762	A(RI)	42.311	1410.35256	0.0000728	A(RI)
**14.458**	481.92851	0.0001927	A(RI)	43.278	1442.58557	0.0001962	A(RI)
**14.495**	483.16184	0.0000694	A(RI)	43.288	1442.9189	0.0000210	A(RI)
**14.51**	483.66183	0.0005624	A(RI)	43.307	1443.55223	0.0000664	A(RI)
**14.555**	485.16182	0.0000030	A(RI)	43.309	1443.6189	0.0001937	A(RI)
**14.671**	489.02844	0.0000297	A(RI)	43.36	1445.31888	0.0000025	A(RI)

**14.746**	491.52842	0.0000005	A(RI)	43.38	1445.98554	0.0005272	A(RI)
**14.789**	492.96174	0.0001047	A(RI)	44.581	1486.01847	0.0002790	A(RI)
**14.812**	493.7284	0.0000221	A(RI)	44.606	1486.8518	0.0001876	A(RI)
**14.834**	494.46172	0.0000080	A(RI)	44.674	1489.11844	0.0056176	A(RI)
**14.868**	495.59504	0.0000003	A(RI)	44.684	1489.45177	0.0000055	A(RI)
**14.936**	497.86169	0.0000029	A(RI)	44.691	1489.6851	0.0024974	A(RI)
**14.947**	498.22835	0.0000908	A(RI)	44.707	1490.21843	0.0013565	A(RI)
**15.207**	506.89493	0.0000557	A(RI)	45.115	1503.8183	0.0038967	A(RI)
**15.212**	507.0616	0.0000000	A(RI)	45.135	1504.48496	0.0001800	A(RI)
**15.219**	507.29493	0.0000018	A(RI)	45.14	1504.65162	0.0000414	A(RI)
**15.232**	507.72826	0.0000079	A(RI)	45.159	1505.28495	0.0180496	A(RI)
**15.291**	509.6949	0.0003560	A(RI)	45.199	1506.61827	0.0013976	A(RI)
**15.335**	511.16156	0.0002954	A(RI)	45.238	1507.91825	0.0047727	A(RI)
**16.05**	534.99465	0.0000014	A(RI)	46.653	1555.08445	0.0104852	A(RI)
**16.072**	535.72798	0.0000000	A(RI)	46.665	1555.48445	0.0086546	A(RI)
**16.243**	541.42792	0.0000000	A(RI)	46.69	1556.31777	0.0044134	A(RI)
**16.268**	542.26124	0.0000001	A(RI)	46.713	1557.08443	0.0004620	A(RI)
**16.327**	544.22789	0.0000000	A(RI)	46.716	1557.18443	0.0001720	A(RI)
**16.36**	545.32788	0.0000373	A(RI)	46.752	1558.38442	0.0002344	A(RI)
**16.499**	549.96117	0.0000184	A(RI)	47.246	1574.85092	0.0011005	A(RI)
**16.542**	551.39449	0.0000177	A(RI)	47.249	1574.95092	0.0000020	A(RI)
**16.57**	552.32781	0.0000039	A(RI)	47.292	1576.38424	0.0000672	A(RI)
**16.598**	553.26113	0.0000204	A(RI)	47.309	1576.9509	0.0001267	A(RI)
**16.665**	555.49445	0.0002486	A(RI)	47.317	1577.21756	0.0015060	A(RI)
**16.722**	557.39443	0.0002040	A(RI)	47.318	1577.25089	0.0008565	A(RI)
**17.999**	599.96067	0.0002952	A(RI)	47.691	1589.6841	0.0001888	A(RI)
**18.014**	600.46066	0.0002536	A(RI)	47.702	1590.05077	0.0001775	A(RI)
**18.082**	602.72731	0.0000511	A(RI)	47.708	1590.25076	0.0000213	A(RI)
**18.098**	603.26063	0.0003165	A(RI)	47.721	1590.68409	0.0000522	A(RI)
**18.104**	603.46063	0.0000471	A(RI)	47.736	1591.18409	0.0000468	A(RI)
**18.132**	604.39396	0.0000021	A(RI)	47.742	1591.38409	0.0000661	A(RI)
**18.736**	624.52709	0.0005293	A(RI)	48.219	1607.28393	0.0004975	A(RI)
**18.772**	625.72708	0.0001645	A(RI)	48.241	1608.01725	0.0021081	A(RI)
**18.784**	626.12707	0.0027300	A(RI)	48.263	1608.75058	0.0005125	A(RI)
**18.796**	626.52707	0.0001748	A(RI)	48.293	1609.75057	0.0003536	A(RI)
**18.84**	627.99372	0.0001304	A(RI)	48.313	1610.41723	0.0006118	A(RI)
**18.88**	629.32704	0.0001441	A(RI)	48.337	1611.21722	0.0018731	A(RI)
**21.32**	710.65956	0.0032000	A(RI)	61.148	2038.24628	0.8031403	A(RI)
**21.326**	710.85956	0.0000910	A(RI)	61.293	2043.07957	0.1308098	A(RI)
**21.351**	711.69288	0.0032300	A(RI)	61.445	2048.14619	0.0030711	A(RI)
**21.361**	712.02621	0.0028200	A(RI)	61.555	2051.81282	0.8045058	A(RI)
**21.382**	712.72621	0.0001387	A(RI)	62.119	2070.61263	0.0728309	A(RI)
**21.406**	713.5262	0.0008240	A(RI)	62.139	2071.27929	0.0747707	A(RI)
**21.627**	720.89279	0.0006997	A(RI)	92.29	3076.30257	0.0002769	A(RI)
**21.641**	721.35945	0.0071400	A(RI)	92.29	3076.30257	0.0083962	A(RI)
**21.649**	721.62612	0.0096200	A(RI)	92.353	3078.40255	0.0057605	A(RI)
**21.656**	721.85945	0.0000542	A(RI)	92.368	3078.90254	0.0006011	A(RI)

**21.676**	722.52611	0.0014700	A(RI)	92.472	3082.36918	0.0006487	A(RI)
**21.694**	723.1261	0.0025900	A(RI)	92.506	3083.5025	0.0005680	A(RI)
**21.734**	724.45942	0.0546000	A(RI)	93.005	3100.13567	0.0000137	A(RI)
**21.775**	725.82608	0.0002760	A(RI)	93.013	3100.40233	0.0002154	A(RI)
**21.779**	725.95941	0.0000007	A(RI)	93.023	3100.73566	0.0001005	A(RI)
**21.827**	727.55939	0.0000106	A(RI)	93.041	3101.33565	0.0013433	A(RI)
**21.872**	729.05938	0.0012700	A(RI)	93.042	3101.36899	0.0057779	A(RI)
**21.89**	729.65937	0.0028700	A(RI)	93.044	3101.43565	0.0035257	A(RI)
**22.562**	752.05915	0.0086100	A(RI)	93.069	3102.26898	0.0000085	A(RI)
**22.612**	753.7258	0.0000000	A(RI)	93.078	3102.56897	0.0014856	A(RI)
**22.642**	754.72579	0.0014800	A(RI)	93.08	3102.63564	0.0000085	A(RI)
**22.652**	755.05912	0.0002956	A(RI)	93.093	3103.06897	0.0006505	A(RI)
**22.7**	756.6591	0.0000000	A(RI)	93.152	3105.03562	0.0007551	A(RI)
**22.78**	759.32574	0.0053000	A(RI)	93.171	3105.66894	0.0008619	A(RI)
**23.31**	776.99223	0.0001560	A(RI)	93.225	3107.46893	0.0001795	A(RI)
**23.339**	777.95889	0.0000071	A(RI)	93.228	3107.56892	0.0002720	A(RI)
**23.903**	796.7587	0.0002486	A(RI)	93.294	3109.7689	0.0003269	A(RI)
**23.972**	799.05868	0.0008496	A(RI)	93.305	3110.13557	0.0011361	A(RI)
**23.975**	799.15868	0.0092700	A(RI)	93.357	3111.86888	0.0000090	A(RI)
**24.007**	800.22533	0.0003687	A(RI)	93.359	3111.93555	0.0030033	A(RI)
**24.692**	823.05844	0.0001308	A(RI)	93.362	3112.03555	0.0022326	A(RI)
**24.705**	823.49177	0.0003208	A(RI)	93.379	3112.60221	0.0011609	A(RI)
**24.71**	823.65843	0.0000008	A(RI)	93.411	3113.66886	0.0069178	A(RI)
**24.743**	824.75842	0.0000013	A(RI)	93.436	3114.50219	0.0042866	A(RI)
**24.754**	825.12508	0.0000759	A(RI)	93.509	3116.9355	0.0000000	A(RI)
**24.765**	825.49175	0.0016800	A(RI)	93.525	3117.46883	0.0000477	A(RI)
**24.853**	828.42505	0.0000007	A(RI)	93.54	3117.96882	0.0002654	A(RI)
**24.878**	829.25837	0.0000140	A(RI)	93.549	3118.26882	0.0002912	A(RI)
**24.904**	830.12503	0.0002236	A(RI)	93.572	3119.03548	0.0002968	A(RI)
**24.928**	830.92502	0.0000018	A(RI)	93.6	3119.9688	0.0003384	A(RI)
**25.081**	836.02497	0.0254800	A(RI)	93.626	3120.83546	0.0000015	A(RI)
**25.087**	836.22497	0.0002729	A(RI)	93.667	3122.20211	0.0001832	A(RI)
**25.137**	837.89162	0.0114500	A(RI)	93.717	3123.86876	0.0026272	A(RI)
**25.151**	838.35828	0.0054400	A(RI)	93.722	3124.03543	0.0068844	A(RI)
**25.232**	841.05826	0.0340100	A(RI)	93.743	3124.73542	0.0000328	A(RI)
**25.256**	841.85825	0.0001038	A(RI)	93.747	3124.86875	0.0087413	A(RI)
**25.312**	843.7249	0.0048400	A(RI)	93.858	3128.56871	0.0030143	A(RI)
**25.369**	845.62488	0.0022800	A(RI)	93.868	3128.90204	0.0025221	A(RI)
**25.595**	853.15814	0.0004503	A(RI)	94.057	3135.20198	0.0000129	A(RI)
**25.623**	854.09146	0.0002490	A(RI)	94.063	3135.40198	0.0000054	A(RI)
**25.637**	854.55812	0.0000883	A(RI)	94.075	3135.80198	0.0000145	A(RI)
**25.646**	854.85812	0.0002289	A(RI)	94.09	3136.30197	0.0000042	A(RI)
**25.662**	855.39145	0.0011800	A(RI)	94.222	3140.70193	0.0000223	A(RI)
**25.687**	856.22477	0.0027300	A(RI)	94.239	3141.26859	0.0000094	A(RI)

A(RI) - 1, x, y, z, x^2, y^2, z^2, xy, xz, yz (symmetry operation); (RI) - Raman and infrared active mode

UV-VIS spectroscopy

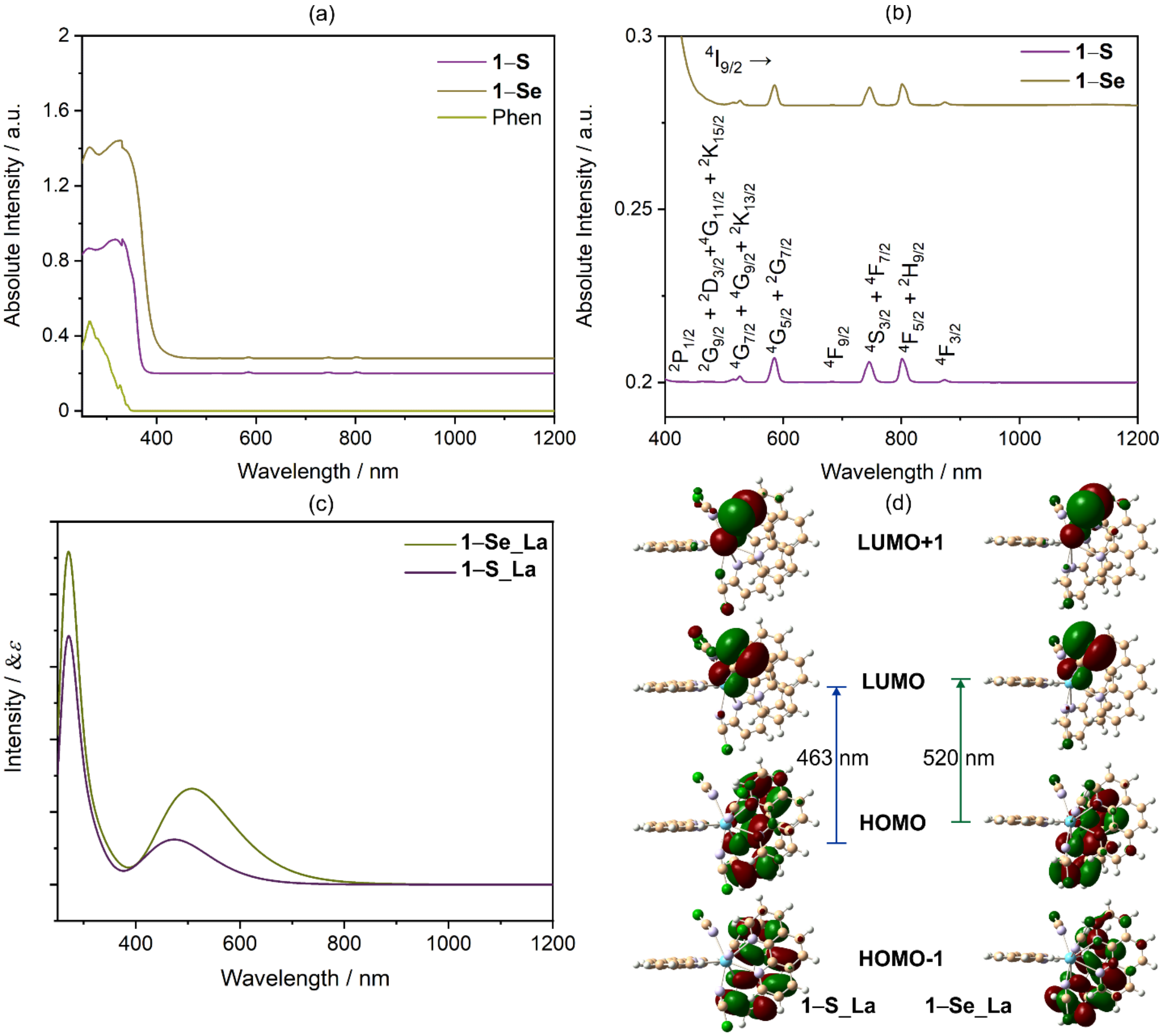

Figure S10. Room-temperature solid-state UV-Vis-NIR absorption (Kubelka-Munk function) spectra of **1**-**S** and **1**-**Se** in (a) 200–1200 nm and (b) 400–1200 nm ranges with indicated assignments, (c) the calculated UV-Vis spectra for **1**-**S** and **1**-**Se** with Nd(III) substituted by La(III) in 200–1200 nm range, and (d) obtained corresponding frontier molecular orbitals.

Emission spectroscopy and thermometric properties

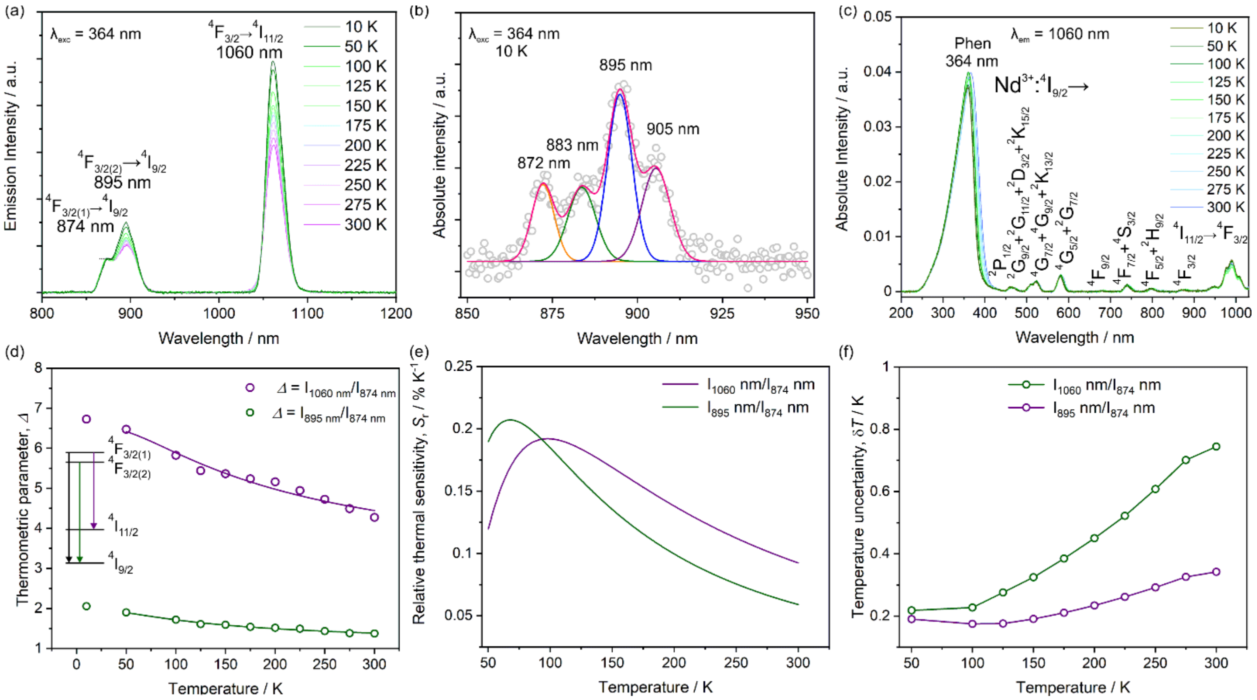

Figure S11. (a) Temperature-dependent emission spectra of **1-Se**. (b) Deconvoluted high-resolution emission spectrum measured at 10 K for **1-Se** with a 364 nm excitation light. (c) Temperature-dependent excitation spectra of **1-Se** were measured for emissive peaks located at 1060 nm. The full characterization of NIR based emissive thermometry for **1-Se** with the Δ being defined as the ratio between peak intensities, evaluated from emission intensities measured at a different temperature: (d) thermometric parameters in the 10–300 K range (magenta and olive circles) along with the fitted line in the range of 50–300 K, (e) the evaluated relative sensitivity at a different temperature from the fitted Δ curve, (f) evaluated temperature uncertainty for different temperatures.

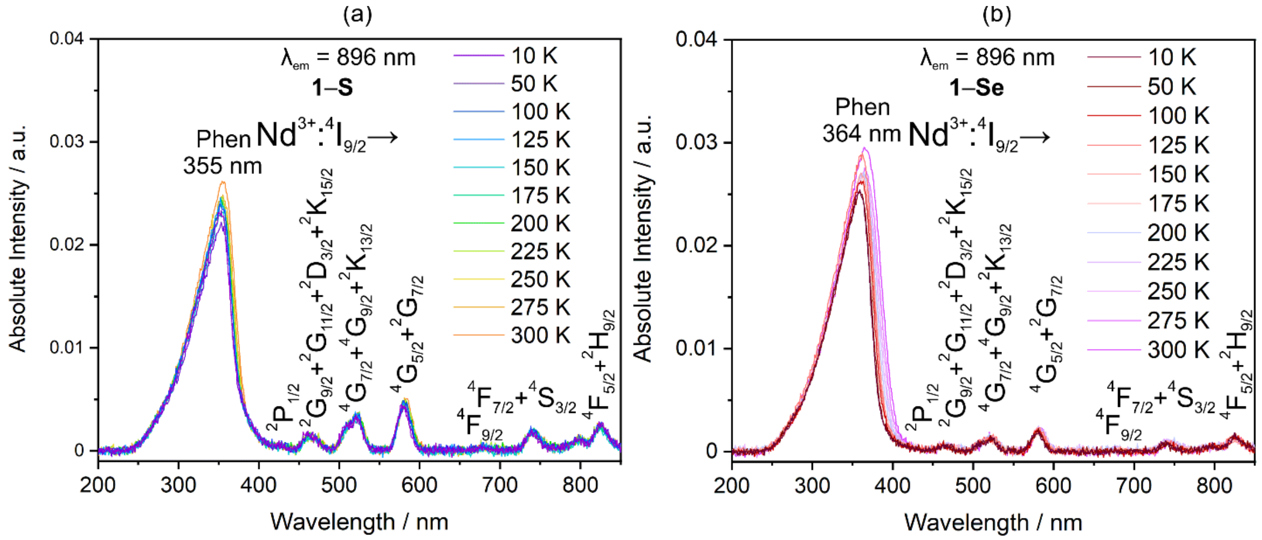

Figure S12. (a, b) Temperature-dependent excitation spectra of **1-S** and**1-Se** recorded for emissive peak centered at 896 nm.

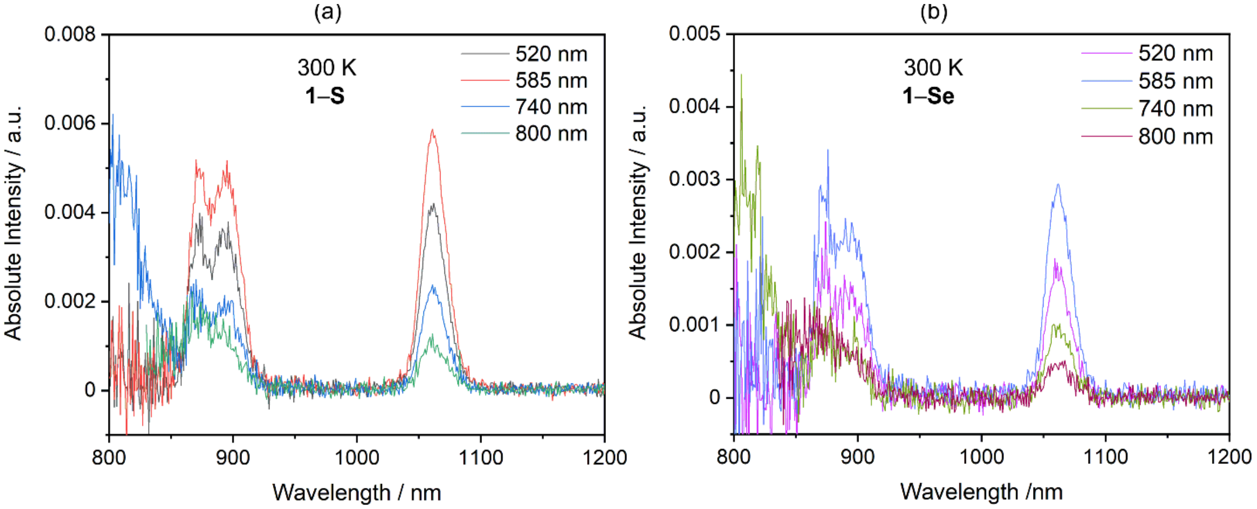

Figure S13. (a, b) Excitation wavelength-dependent near-infrared emission spectra collected at 300 K for **1-S** and **1-Se**.

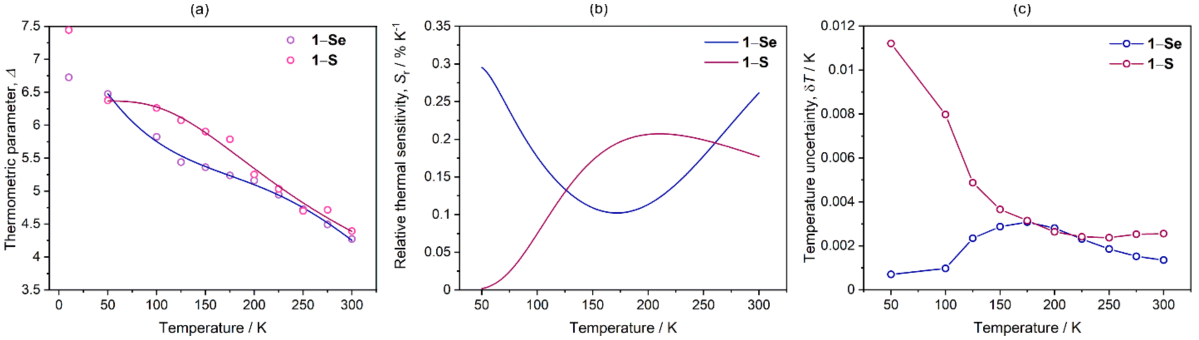

Figure S14. (a) Thermometric parameters measured in the 10–300 K range along with the fitted line in the range of 50–300 K for peaks positioned near 1060 nm and 874 nm. (b) Evaluated relative sensitivity at a different temperature from the fitted *Δ* curve. (c) Evaluated temperature uncertainty for different temperatures. Equation S1 was used to fit panel (a).

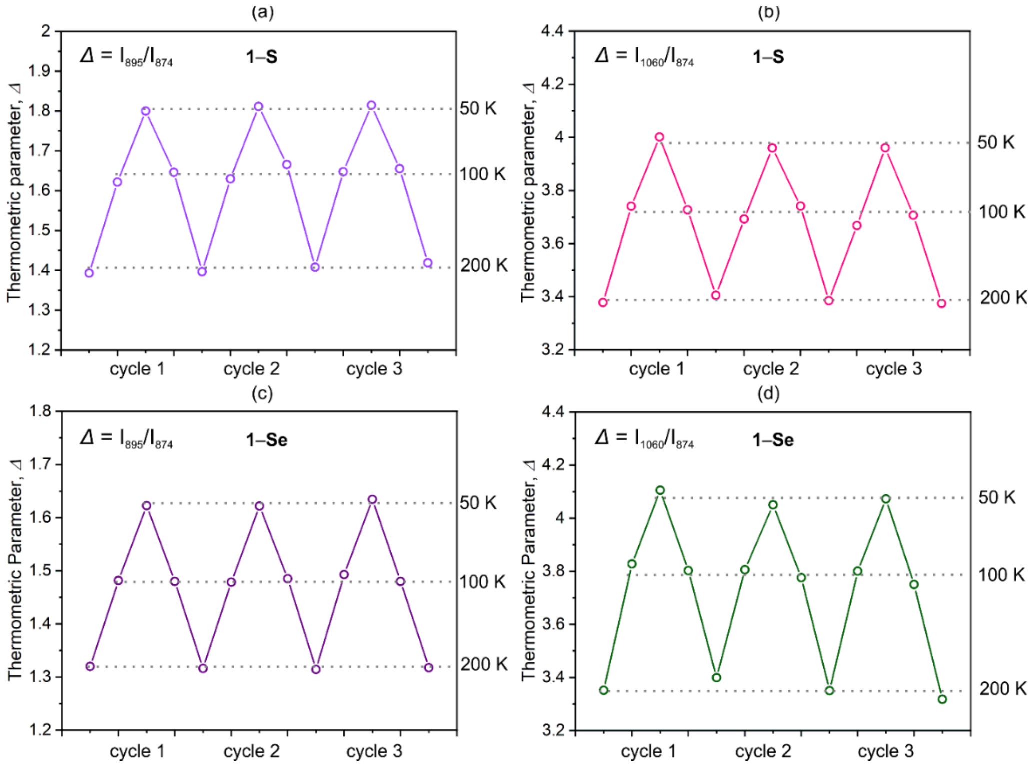

Figure S15. Three cycles of repeatability data of thermometric parameters measured for three temperatures 50, 100, and 200 K for indicated thermometric parameters.

Table S6: CASSCF–SO results for the $^4F_{9/2}$ ground term of **1-S**.

Energy (cm^{-1})	$g_\perp$	$g_\parallel$	CF wave function composition	$\langle \hat{J}_z \rangle$
**0.0**	1.88	4.32	16.0 % |±1/2⟩ + 3.4 % |∓3/2⟩ + 8.4 % |±5/2⟩+19.1 % |±7/2⟩ + 49.7 % |±9/2⟩	±2.162
**66.5**	1.07	2.82	26.1 % |±1/2⟩ + 28.6 % |∓3/2⟩ + 24.3 % |±5/2⟩+6.7 % |±7/2⟩ + 13.7 % |±9/2⟩	±0.518
**122.4**	1.23	3.57	7.2 % |±1/2⟩ + 20.0 % |∓3/2⟩ + 16.5 % |±5/2⟩+44.1 % |±7/2⟩ + 12.3 % |±9/2⟩	±1.571
**154.2**	1.94	3.63	33.5 % |±1/2⟩ + 27.5 % |∓3/2⟩ + 20.3 % |±5/2⟩+6.1 % |±7/2⟩ + 12.6 % |±9/2⟩	±1.042
**210.3**	1.96	4.55	16.9 % |±1/2⟩ + 16.8 % |∓3/2⟩ + 29.3 % |±5/2⟩+19.0 % |±7/2⟩ + 18.0 % |±9/2⟩	±1.376

Table S7: CASSCF–SO results for the $^4F_{9/2}$ ground term of **1-Se**.

Energy (cm^{-1})	$g_\perp$	$g_\parallel$	CF wave function composition	$\langle \hat{J}_z \rangle$
**0.0**	1.71	4.46	16.0 % |±1/2⟩ + 6.8 % |∓3/2⟩ + 9.5 % |±5/2⟩+17.2 % |±7/2⟩ + 5.9 % |±9/2⟩	±2.232
**68.9**	1.09	2.78	23.9 % |±1/2⟩ + 29.6 % |∓3/2⟩ + 26.2 % |±5/2⟩+7.2 % |±7/2⟩ + 13.0 % |±9/2⟩	±0.508
**125.4**	1.28	3.53	5.1 % |±1/2⟩ + 21.5 % |∓3/2⟩ + 19.8 % |±5/2⟩+43.0 % |±7/2⟩ + 10.4 % |±9/2⟩	±1.512
**159.0**	1.78	3.83	36.9 % |±1/2⟩ + 27.3 % |∓3/2⟩ + 17.7 % |±5/2⟩+6.9 % |±7/2⟩ + 11.2 % |±9/2⟩	±0.934
**213.0**	1.88	4.62	18.1 % |±1/2⟩ + 14.7 % |∓3/2⟩ + 27.8 % |±5/2⟩+23.7 % |±7/2⟩ + 15.6 % |±9/2⟩	±1.349

Mott–Seitz model for two electronic transitions[2]

$$\Delta(T) = \frac{\Delta_{10}}{[1+\alpha_1 \exp\left(-\frac{\Delta E_1}{k_B T}\right)+\alpha_2 \exp\left(-\frac{\Delta E_2}{k_B T}\right)]} \quad \textbf{(S1)}$$

where Δ_{10} is the thermometric parameter at T = 0 K, α_1 and α_2 is the ratio between nonradiative (W_0 at T = 0 K) and radiative (W_R) rates are W_0/W_R for 1060 and 874 nm, respectively, and ΔE_1, ΔE_2 is the activation energies of the nonradiative channels for 1060 and 874 nm transition.

Table S8. List of the fitting parameters from Δ vs. temperature plot for **1-S** and **1-Se** fitted with Equation 1 given in the manuscript and Equation S1.

Compounds	parameter	*Δ*(895/874)	*Δ*(1060/874)	*Δ*(1060/874)_mott Seitz model
**1-S**	A	2.131	6.402	
	Δ_0	-2.091	-7.740	
	$\Delta E_0/k_B$	374.529	400.361	
	Δ_{10}			6.368
	$\Delta E_1/k_B$			512.698
	α_1			1.992
	$\Delta E_2/k_B$			512.665
	α_2			0.500
	$R^2(COD)$	0.987	0.983	0.991
**1-Se**	A	1.967	6.525	
	Δ_0	-0.887	-3.761	
	$\Delta E_0/k_B$	126.118	177.695	
	Δ_{10}			7.001
	$\Delta E_1/k_B$			1527.857
	α_1			36.868
	$\Delta E_2/k_B$			98.964
	α_2			0.584
	$R^2(COD)$	0.984	0.965	0.991

Table S9. The summary of maximum and minimum calculated relative thermal sensitivity, S_r, and temperature uncertainty, ΔT, for **1-S** and **1-Se** at the indicated temperature.

Compound	parameters	*Δ*(895/874)	*Δ*(1060/874)	*Δ*(1060/874)_mott Seitz model
**1-S**	S_r (max., % K^{-1})	0.168@230 K	0.207@300 K	0.207@200 K
	S_r (min., % K^{-1})	0.006@50 K	0.008@50 K	0.003@50 K
	ΔT (max., K)	1.148@50 K	1.151@50 K	0.011@50 K
	ΔT (min., K)	0.319@250 K	0.344@250 K	0.002@250 K
**1-Se**	S_r (max., % K^{-1})	0.207@170 K	0.192@100 K	0.295@50 K
	S_r (min., % K^{-1})	0.0509@300 K	0.092@300 K	0.102@170 K
	ΔT (max., K)	0.341@300 K	0.744@300 K	0.003@175 K
	ΔT (min., K)	0.227@100 K	0.175@100 K	0.0008@50 K

References to Supporting Information

(1) M. Llunell, D. Casanova, J. Cirera, J. Bofill, P. Alemany, S. Alvarez, M. Pinsky and D. Avnir, *SHAPE v.2.1.* Program for the Calculation of Continuous Shape Measures of Polygonal and Polyhedral Molecular Fragments, University of Barcelona: Barcelona, Spain, 2013.

(2) Kumar, K.; Chorazy, S.; Nakabayashi, K.; Sato, H.; Sieklucka, B.; Ohkoshi, S.I. TbCo and Tb 0.5 Dy 0.5 Co layered cyanido-bridged frameworks for construction of colorimetric and ratiometric luminescent thermometers. *J. Mater. Chem. C* **2018**, *6*, 8372–8384
